# Multi-species assessment of propranolol pharmacokinetics via minimal and whole-body PBPK modeling

**DOI:** 10.3389/fphar.2026.1933766

**Published:** 2026-09-10

**Authors:** Minsoo Lee, Min Ji Ji, Yoo-Seong Jeong, Wooin Lee

**Affiliations:** College of Pharmacy and Research Institute of Pharmaceutical Sciences, Seoul National University, Seoul, Republic of Korea

**Keywords:** allometric scaling, interspecies scaling, minimal and whole-body models, physiologically-based pharmacokinetic modeling, propranolol

## Abstract

Propranolol is a widely-used β-adrenergic blocker with extensive pharmacokinetic (PK) data across multiple species, rendering it appropriate for comparing various interspecies scaling methodologies. The present study aimed to apply a quantitative interspecies PK modeling framework for propranolol using literature-derived datasets from seven mammalian species, namely, mouse, rat, rabbit, cat, dog, human, and horse. Conventional allometric analyses indicated body weight-dependent relationships for both clearance and steady-state volume of distribution. For the top-down minimal physiologically-based pharmacokinetic (mPBPK) modeling approach, the model incorporated two peripheral tissue compartments governed by shared distribution parameters across different species. The simultaneous fitting of intravenous (IV) propranolol PK data from seven species to the mPBPK model produced parameter estimates that reasonably captured the observed plasma PK data. Subsequent individual fitting of IV and oral propranolol PK data for each species adequately estimated additional absorption-related parameters. To improve physiological interpretability, top-down parameter estimates were aligned with bottom-up whole-body PBPK parameters via tissue-lumping principles. A unitless product-ratio plot further provided species-dependent kinetic distinctions on the biological half-lives of propranolol. Finally, translational performance was evaluated by scaling preclinical datasets, where the mPBPK model derived from three common preclinical species provided reasonable predictions of human IV PK profiles. Collectively, these findings provide a clearer physiological interpretation of propranolol disposition across species. Building upon the previous efforts using mPBPK modeling, the current study demonstrates a more mechanistically informed, refined workflow for interspecies scaling and translational human PK prediction applicable to drug candidates.

## 1 Introduction

During drug development, accurately predicting human pharmacokinetics (PK) remains a major challenge, particularly when preclinical studies provide limited *in vivo* plasma data. While allometric scaling is widely used, its empirical nature limits physiological interpretability. More importantly, it poses challenges when preclinical PK data do not conform to traditional allometric relationships due to substantial interspecies differences (Ward and Smith, 2004a, b). As such, it is advisable to supplement allometric scaling with approaches informed by mechanistic understanding.

Physiologically-based pharmacokinetic (PBPK) modeling is frequently used for mechanistic interspecies extrapolation. Whole-body PBPK (WB-PBPK) models can incorporate anatomical and physiological differences across species, but require numerous tissue-specific parameters, which are resource-intensive to determine experimentally. As a practical alternative, the minimal PBPK (mPBPK) model has been proposed (Cao and Jusko, 2012), which retains key physiological features while substantially reducing model complexity. This approach facilitates physiologically interpretable modeling primarily based on plasma data and has been applied to interspecies PK analysis of several drugs, including moxifloxacin (Cao and Jusko, 2012), monoclonal antibodies (Zhao et al., 2015), metformin (Jeong and Jusko, 2021), dexamethasone (Song and Jusko, 2021), metronidazole (Kir and Jusko, 2025), betamethasone (Zhang and Jusko, 2025), methylprednisolone (Yu and Jusko, 2025), levamisole (Cheng et al., 2026), and butorphanol (Saeed and Jusko, 2026). These studies provide a foundational workflow to guide the implementation of the interspecies mPBPK modeling approach. However, prior interspecies mPBPK modeling efforts have relied primarily on plasma PK data. For drugs with experimentally determined tissue distribution data, comparing top-down mPBPK estimates with bottom-up WB-PBPK models may enhance physiological interpretability and provide additional mechanistic insight.

In mPBPK modeling, additional conceptual clarification can enhance mechanistic understanding of PK behavior. For instance, peripheral tissue compartments lack explicit anatomical assignments, and the model parameters estimated in the differential equation domain are not readily linked to plasma PK characteristics in the analytical solution domain. A tissue lumping principle was proposed to align mPBPK and WB-PBPK models in rats (Jeong et al., 2022a, b). The overall shape of the plasma PK profile was found to depend on the relative magnitudes of the mean residence time in the body ( $MRT_{B}$ ) and the mean transit time through the kinetically largest tissue ( $MTT_{\max}$ ), expressed as the ratio $K_{\det}=MTT_{\max}/MRT_{B}$ (Jeong and Jusko, 2022b; Jeong and Jusko, 2023). In addition, the product of $MRT_{B}$ and the terminal slope ( $\lambda_{z}$ ), defined as $P_{\det}=MRT_{B}\cdot \lambda_{z}$ , was proposed as a useful criterion for identifying the limiting condition of the biological half-life ( $T_{1/2}$ ) in PBPK systems. However, these theoretical frameworks have not yet been applied to interspecies PK analysis.

Propranolol is a non-selective β-adrenergic receptor antagonist used for the treatment of cardiovascular diseases (e.g., hypertension, myocardial infarction) as well as neurological conditions (e.g., migraine and tremor) since its approval by the United States Food and Drug Administration in 1964. Propranolol has been extensively studied across multiple species and experimental systems, yielding a comprehensive collection of *in vivo* and *in vitro* PK data. *In vivo*, propranolol exhibits extensive tissue distribution, with the steady-state volume of distribution ( $V_{SS}$ ) ranging from 1.72 L/kg in cats (Jacobs et al., 1997) to 8.67 L/kg in rabbits (Al-Meshal et al., 1993), and is characterized by a high hepatic extraction ratio ( ER ) in rats (Jones et al., 1985). *In vitro*, propranolol is recognized for its high membrane permeability and is frequently used as a reference compound in cell- or membrane-based permeability assays (Mendes et al., 2015), aligning with its high human intestinal permeability (Lennernas, 2007). Despite the availability of extensive cross-species and cross-system datasets, previous studies have largely relied on allometric scaling (Lave et al., 1997; Mahmood, 2000) or single-species PBPK modeling, primarily focusing on humans (Levitt, 2002) or rats (Poulin and Theil, 2002; Cheung et al., 2018). Taken together, propranolol represents an appropriate model compound for an interspecies PK modeling approach that integrates available *in vivo* and *in vitro* data via top-down or bottom-up approaches.

The present study compiled propranolol PK data across seven species and performed interspecies mPBPK modeling using complementary top-down and bottom-up approaches. Unlike previous interspecies mPBPK analyses, which relied primarily on plasma PK data, the current study leveraged experimentally determined tissue distribution data for propranolol (Rodgers et al., 2005), enabling comparison of the top-down mPBPK estimates with bottom-up WB-PBPK models and identification of tissue constituents comprising the lumped compartments across species (Jeong et al., 2022a, b). We also applied the unitless product-ratio plot to the interspecies dataset to evaluate whether $\lambda_{z}$ was primarily governed by $MTT_{\max}$ or $MRT_{B}$ across species (Jeong and Jusko, 2022b). Finally, the translational performance of this approach was assessed by predicting human PK profiles using parameters derived from three preclinical species. Overall, the current study provides a mechanistically informed workflow for interspecies scaling and translational human PK prediction, incorporating physiological interpretation of tissue disposition and limiting-condition analysis, with potential applicability to other drug candidates.

## 2 Methods

### 2.1 Data acquisition

The overall workflow of the interspecies PK analysis is presented in Supplementary Figure S1. The propranolol PK data across multiple species were identified through a systematic literature search using PubMed and Google Scholar, combining the keywords “propranolol,” “pharmacokinetics,” “distribution,” and “species.” Publications were screened according to the following criteria: (i) full-text accessibility, (ii) studies conducted in healthy subjects, and (iii) extractable plasma concentration-time data. When necessary, reference tracing was additionally performed to identify relevant datasets, particularly for species with limited data availability. All collected datasets are summarized in Supplementary Table S1.

Graphically presented PK data were digitized using WebPlotDigitizer (v5, https://automeris.io). To ensure data reliability, non-compartmental analysis (NCA) was performed using Phoenix WinNonlin® (Build 8.1.0.3530, Certara, Princeton, NJ, United States), and the resulting PK parameters, including the total clearance ( $CL_{tot}$ ) and the steady-state volume of distribution ( $V_{SS}$ ), were compared with the reported values. Datasets with a less than 1.5-fold difference between the reported and NCA-calculated values were considered suitable and retained for subsequent analysis.

### 2.2 Interspecies allometric scaling

Plasma PK profiles following intravenous (IV) and oral administration across seven mammalian species (mouse, rat, rabbit, cat, dog, human, and horse) were compiled from the literature (Supplementary Table S1). When multiple studies were available for a given species, representative datasets were selected based on (i) sufficient sampling density, (ii) adequate characterization of the terminal phase, and (iii) analytical reliability, as summarized in Table 1. For rats, IV and oral PK datasets showed substantial inter-study variability, as evidenced by dose-normalized systemic exposure [maximum plasma concentration ( $C_{\max}/Dose$ ) and area under the curve from time 0 to infinity ( AUC/Dose ) values] that differed by ∼100-fold even at the same dose (Supplementary Figure S2). Since the factors underlying this variability could not be identified, six datasets that deviated substantially from the remainder were excluded from the analysis (Table 1). For humans, the five datasets reporting both IV and oral PK were selected for model development, allowing oral bioavailability ( F ) to be evaluated using data obtained within the same study.

**TABLE 1:** Summary of the representative PK datasets of propranolol after intravenous (IV) and oral (PO) administration in seven species analyzed in the current study.

Species	Strain/study population	Sex	Body weight (kg)	Dosing route	Dose	CL (mL/min/kg)	CL/F (mL/min/kg)	$V_{SS}$ (L/kg)	References
Mouse	Swiss-Webster	M, F	0.02–0.025	IV^a^	16 mg/kg	179^b^	—	12.0	(Levy et al., 1976)
Mouse	CD/1	—	—	PO	30 mg/kg	—	311	—	(Chen et al., 2006)
Rat	Sprague-Dawley	M	—	IV,^a^ PO	3, 10 mg/kg	30.7	290^b^	8.37	(Lee and Ku, 1999)
Rat	Sprague-Dawley	M, F	0.220	PO	20 mg/kg	—	398	—	(Al Shaker et al., 2017)
Rat	Wistar	M	0.230–0.270	PO	2.5, 10 mg/kg	—	547–229	—	(Yoshigae et al., 1998)
Rat	Wistar	M	0.443	PO	30 mg/kg	—	309^b^	—	(Laethem et al., 1994)
Rat	Wistar	—	0.300	IV, PO	1.5, 15 mg/kg	26.9	475–510	—	(Vercruysse et al., 1993)
Rabbit	New-Zealand	M	3	PO	50 mg/kg	—	19,500^b^	—	(Laethem et al., 1995)
Rabbit	New-Zealand White	M	3–5	IV^a^	1 mg/kg	85.5	—	8.67	(Al-Meshal et al., 1993)
Cat	—	M	—	IV^a^	0.5–1 mg/kg	43.4	—	3.37	(Arendt et al., 1984)
Cat	—	F	—	IV	0.5 mg/kg	28	102	1.72	(Jacobs et al., 1997)
				PO	5 mg/kg				
Dog	Mongrel	M, F	10–20	IV infusion^a^	0.1 mg/kg/min	14.8^b^	—	3.09^b^	(Walle et al., 1989)
Dog	Mongrel	M	25–30	PO	5 mg/kg	—	632^b^	—	(Laethem et al., 1995)
Human	Healthy	M, F	—	IV^a^	1 mg	1,190 mL/min	3,580 mL/min	4.4	(Taegtmeyer et al., 2014)
				PO	40 mg				
Human	Healthy	—	25% within IBW	IV	10 mg	1,120 mL/min^b^	—	301 L^b^	(Bleske et al., 1995)
Human	Healthy	M, F	62–92	IV	0.1 mg/kg	10.7	—	2.72	(Olanoff et al., 1984)
Human	Healthy	M, F	79.4	IV	10 mg	11.6	99.5–160	3.72	(Borgstrom et al., 1981)
				PO	40 mg, 80 mg, 120 mg				
Human	Healthy	M	64.5	IV infusion	0.1 mg/kg	1,060 mL/min^b^	—	344 L^b^	(Gomeni et al., 1977)
				PO	10 mg, 20 mg, 40 mg				
Horse	Gelding	M	490–600	IV^a^	0.2 mg/kg	22.9	813	3.05	(Aramaki et al., 2000)
				PO	1 mg/kg				

The table includes IV and PO data expressed in terms of propranolol racemate. Other PK datasets involving stereoselectivity or non-healthy subjects are listed in Supplementary Table S1.

^a^ Data used for simultaneous model fitting.

^b^ Calculated using digitized data.

Using these representative datasets, allometric scaling was conducted according to the power function $Y=a\cdot BW^{b}$ (Boxenbaum, 1982), where Y represents PK parameters ( $CL_{tot}$ and $V_{SS}$ ) and BW is body weight. Linear regression was applied to the log-transformed data to determine the allometric coefficient ( a ) and the exponent ( b ).

### 2.3 mPBPK model construction

**FIGURE 1:**
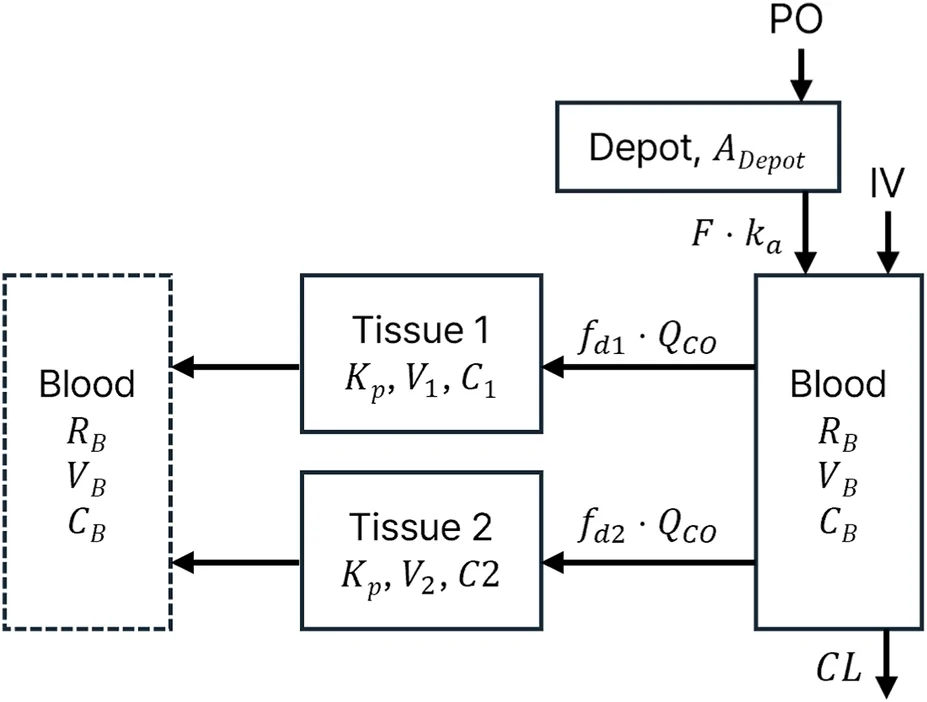
Structure of the mPBPK model for propranolol. The model consists of a blood compartment and two tissue compartments (Tissues 1 and 2). Model assumptions and symbols are described in the text. The volumes of the two tissue compartments are defined as follows: $V_{1}=\left(BW-V_{B}\right)\cdot f_{T1}$ and $V_{2}=\left(BW-V_{B}\right)\cdot f_{T2}$ .

The mPBPK model consisted of a blood compartment and two lumped tissue compartments (Tissues 1 and 2; Figure 1) as previously reported (Cao and Jusko, 2012). The governing equations are presented as Equations 1–3:

$$V_{B}{\cdot R}_{B}\cdot \frac{dC_{p}}{dt}=Input-Q_{1}\cdot R_{B}\cdot \left(C_{p}-\frac{C_{1}}{K_{p}}\right)-Q_{2}\cdot R_{B}\cdot \left(C_{p}-\frac{C_{2}}{K_{p}}\right)-CL\cdot C_{p}$$ (1)

$$V_{1}\cdot \frac{dC_{1}}{dt}=Q_{1}\cdot R_{B}\cdot \left(C_{p}-\frac{C_{1}}{K_{p}}\right)$$ (2)

$$V_{2}\cdot \frac{dC_{2}}{dt}=Q_{2}\cdot R_{B}\cdot \left(C_{p}-\frac{C_{2}}{K_{p}}\right)$$ (3)

where $C_{p}$ ​ is the plasma concentration, $R_{B}$ is the blood-to-plasma partition coefficient fixed at 0.85 across species (Levitt, 2002), $C_{1}$ and $C_{2}$ ​ denote drug concentrations in Tissues 1 and 2, and $K_{p}$ is the tissue-to-plasma partition coefficient assumed to be identical for the two tissue compartments because separate compartment-specific $K_{p}$ values are not independently identifiable from plasma data (Jeong and Jusko, 2023). Drug elimination was assumed to occur from the blood via systemic clearance ( CL ), which was considered species-specific. Species-specific physiological parameters, including blood volume ( $V_{B}$ ), cardiac output ( $Q_{CO}$ ), and hepatic blood flow ( $Q_{H}$ ) were obtained from the literature (Supplementary Table S2).

Given the high lipophilicity (log P = 3.48) and the apparent permeability coefficient (0.88 × 10^−6^ cm/s) of propranolol (Jeong et al., 2017), perfusion-limited distribution was assumed in the mPBPK model, as in the previous study (Poulin and Theil, 2002). The fractional distribution parameters $f_{d1}$ and $f_{d2}$ were used to define the distributional clearances ( $Q_{1}$ and $Q_{2}$ ) such that $Q_{1}=Q_{CO}{\cdot f}_{d1}$ and $Q_{2}=Q_{CO}\cdot f_{d2}$ under the constraint that $f_{d1}+f_{d2}=1$ . The anatomical tissue volumes $V_{1}$ and $V_{2}$ were defined based on the volume fraction of Tissue 1 ( $f_{T1}$ ) and Tissue 2 ( $f_{T2}=1-f_{T1}$ ), expressed as Equations 4, 5:

$$V_{1}=\left(BW-V_{B}\right)\cdot f_{T1}$$ (4)

$$V_{2}=\left(BW-V_{B}\right)\cdot f_{T2}$$ (5)

Initial conditions for Equations 1–3 were set to zero, except for IV bolus administration where the initial plasma concentration was set to $Dose/\left(V_{B}\cdot R_{B}\right)$ and the Input term in Equation 1 was set to zero. For IV infusion, the Input term was set to a constant infusion rate over the dosing interval. For oral administration, absorption was described using a first-order input from a depot compartment ( $A_{depot}$ , Figure 1), described as Equations 6, 7:

$$\frac{dA_{Depot}}{dt}=-k_{a}\cdot A_{Depot}$$ (6)

$$Input=F\cdot k_{a}\cdot A_{Depot}$$ (7)

where $k_{a}$ is the first-order absorption rate constant, and F denotes the oral bioavailability. The initial condition for Equation 6 was set to the oral dose.

### 2.4 Stepwise mPBPK model fitting and parameter estimation

Model fitting was conducted in two steps, as depicted in Supplementary Figure S1. First, the representative IV data from seven species (listed in Table 1) were simultaneously fitted to the mPBPK model. Distribution parameters (i.e., $f_{d1}$ , $f_{T1}$ , $K_{p}$ ) were estimated as shared parameters across species, while CL values were estimated for each species. Second, the representative IV and oral data for each species (listed in Table 1) were fitted individually, while additionally estimating F and $k_{a}$ .

The mPBPK model fitting was performed using ADAPT 5 software (D’Argenio et al., 2009), with the maximum likelihood method. The variance model was defined as Equation 8:

$$V_{i}={\left(\sigma_{1}+\sigma_{2}\cdot Y_{i}\right)}^{2}$$ (8)

where $V_{i}$ is the variance of the *i*th data point, $Y_{i}$ is the corresponding model prediction, and $\sigma_{1}$ and $\sigma_{2}$ are the variance model parameters. Model performance was evaluated by visual inspection of the observed and model-predicted PK profiles, and parameter precision was expressed as the coefficient of variation (CV%). The model code used for simultaneous fitting across seven species is provided in the Supplementary Material. Figures were generated using GraphPad Prism (v11.0.0, GraphPad Software, Boston, MA, United States).

### 2.5 Assessment of the limiting condition of the biological half-lives of propranolol across seven species

In mammillary PBPK systems under the assumptions of linear PK and elimination from the blood, the terminal slope ( $\left.\lambda_{z}\right)$ is governed by two time scales: The mean residence time in the body ( $MRT_{B}$ ) and the mean transit time through the kinetically largest tissue ( $MTT_{\max}$ ). When the overall body residence time is limiting, *λ_z_* approaches *1/MRT_B_*, whereas when the transit through the kinetically largest tissue is limiting, *λ_z_* approaches $1/MTT_{\max}$ . The theoretical range of the terminal slope $\lambda_{z}$ is given by Equation 9 (Jeong and Jusko, 2022b):

$$\frac{1}{MRT_{B}+MTT_{\max}}<\lambda_{z}<\min\left(\frac{1}{MRT_{B}},\frac{1}{MTT_{\max}}\right)$$ (9)

Defining $K_{\det}=MTT_{\max}/MRT_{B}$ and $P_{\det}=MRT_{B}\cdot \lambda_{z}$ gives the corresponding dimensionless relationship (Equation 10):

$$\frac{1}{1+K_{\det}}<P_{\det}<\min\left(1,\frac{1}{K_{\det}}\right)$$ (10)

where $K_{\det}$ describes the relative magnitude of the two time scales, whereas $P_{\det}$ evaluates the proximity of $\lambda_{z}$ to $1/MRT_{B}$ . The corresponding quantity for evaluating the proximity of $\lambda_{z}$ to $1/MTT_{\max}$ is $P_{\det}\cdot K_{\det}$ , which is equivalent to $MTT_{\max}\cdot \lambda_{z}$ and is bounded by (Equation 11):

$$\frac{K_{\det}}{1+K_{\det}}<P_{\det}\cdot K_{\det}<\min\left(K_{\det},1\right)$$ (11)

A product approaching 1 indicates that $\lambda_{z}$ is kinetically associated with the reciprocal of the corresponding time constant. Because both products, $P_{\det}$ and $P_{\det}\cdot K_{\det}$ , are bounded above by 1 under Equations 10, 11, a value greater than 0.5 indicates association within a factor of two. Accordingly, $P_{\det}>0.5$ supports an $MRT_{B}$ -associated terminal slope, whereas $P_{\det}\cdot K_{\det}>0.5$ supports an $MTT_{\max}$ -associated terminal slope. When both exceed 0.5, $\lambda_{z}$ is within a factor of two of both reciprocal time constants, indicating an intermediate rather than a uniquely $MRT_{B}$ - or $MTT_{\max}$ -limited condition. The values of $P_{\det}$ and $P_{\det}{\cdot K}_{\det}$ may differ across species, resulting in $MRT_{B}$ *-*associated, $MTT_{\max}$ -associated, or intermediate terminal disposition.

To assess whether $\lambda_{z}$ values estimated from propranolol PK profiles were closely associated with $1/MRT_{B}$ or $1/MTT_{\max}$ across species, $MRT_{B}$ and MTT values through Tissues 1 and 2 ( $MTT_{1}$ and $MTT_{2}$ ) were calculated from the fitted mPBPK model parameters using Equations 12–14:

$$MRT_{B}=\frac{V_{SS}}{CL}=\frac{V_{1}\cdot K_{p}+V_{2}\cdot K_{p}}{CL}$$ (12)

$$MTT_{1}=\frac{V_{1}\cdot K_{p}}{Q_{CO}\cdot f_{d1}\cdot R_{B}}$$ (13)

$$MTT_{2}=\frac{V_{2}\cdot K_{p}}{Q_{CO}\cdot f_{d2}\cdot R_{B}}$$ (14)

where $MTT_{2}$ , which is defined as greater than $MTT_{1}$ , corresponds to $MTT_{\max}$ in Equations 9, 11. The model-derived $\lambda_{z}$ values were obtained from numerically simulated PK profiles using the fitted parameters for the seven species.

Mathematical computations and visualizations were executed using Python (version 3.13) with the NumPy and Matplotlib (version 3.10.1) libraries. The theoretical upper and lower bounds (Equation 10) were used to situate the calculated $P_{\det}$ and $K_{\det}$ values within this product-ratio space. Based on the theoretical boundaries of the unitless product-ratio plot, a threshold value of 0.5 was used to identify whether $\lambda_{z}$ was more closely associated with $1/MRT_{B}$ or $1/MTT_{\max}$ . In addition, the previously proposed $K_{\det}$ threshold of 0.3 for discriminating the number of tissue compartments in mPBPK models was used to evaluate the applicability of the two-tissue structure across species (Jeong et al., 2022a, b).

### 2.6 Identification of anatomical tissue constituents in the mPBPK model via reconciliation with WB-PBPK models for propranolol in mice, rats, and humans

To provide a mechanistic basis for the two peripheral compartments (Tissues 1 and 2) in the mPBPK model, WB-PBPK models for propranolol were developed for mice, rats, and humans using relevant physiological parameters (Supplementary Table S3). In rats, literature-reported tissue distribution data for major organs (Rodgers et al., 2005) enabled data-driven model construction, analogous to previous approaches (Poulin and Theil, 2002; Cheung et al., 2018). The corresponding WB-PBPK models for mice and humans were subsequently developed using the species-specific physiological parameters. Briefly, the WB-PBPK model consisted of the blood and nine tissue compartments [i.e., skin, muscle, adipose, bone, brain, heart, kidney, lung, and the splanchnic (SPL) compartment]. Tissue-specific $K_{p}$ values were determined based on Supplementary Equation S1 for rats, Supplementary Equation S9 for mice, and Supplementary Equation S10 for humans (Supplementary Table S4), while the CL values were fixed as the mPBPK model estimates (see Results). The detailed WB-PBPK structure (Supplementary Figure S3), governing equations (Supplementary Equations S7, S8), and model development procedures are provided in the Supplementary Material.

To identify the anatomical tissue constituents corresponding to the two peripheral compartments (Tissues 1 and 2) in the mPBPK model structure, the developed WB-PBPK models were reduced using the tissue lumping principle (Jeong et al., 2022a, b). The MTT values through the anatomical tissues were calculated as:

$$MTT=\frac{V_{T}\cdot K_{p}}{Q_{T}\cdot R_{B}}$$ (15)

where $V_{T}$ and $Q_{T}$ ​ are the anatomical tissue volume and the blood flow to the tissue (Supplementary Table S3). When tissues were ranked in descending order of their MTT values, tissues exhibiting relatively long MTT s could be grouped into the slowly equilibrating group (SEG, corresponding to Tissue 2 in the mPBPK model), whereas the remaining tissues were assigned to the rapidly equilibrating group (REG, corresponding to Tissue 1 in the mPBPK model). The aggregated distribution volumes and distributional clearances for the SEG ( $\sum_{i\in SEG}V_{T,i}{\cdot K}_{p,i}$ and $\sum_{i\in SEG}Q_{T,i}{\cdot R}_{B}$ ) and the REG ( $\sum_{i\in REG}V_{T,i}\cdot K_{p,i}$ and $\sum_{i\in REG}Q_{T,i}{\cdot R}_{B}$ ) in the reduced model were then compared with the top-down mPBPK parameter estimates for Tissue 1 ( $V_{1}\cdot K_{p}$ and $Q_{CO}{\cdot f}_{d1}\cdot R_{B}$ ) and Tissue 2 ( $V_{2}\cdot K_{p}$ and $Q_{CO}\cdot f_{d2}\cdot R_{B}$ ). Model calculations were also compared between the reduced model (Supplementary Equations S11, S13) and the top-down mPBPK model (Equations 1–3).

### 2.7 Application of the interspecies mPBPK modeling for human PK prediction of propranolol

We evaluated whether human propranolol PK profiles following IV administration could be reasonably predicted using mPBPK parameters derived from three commonly used preclinical species (mice, rats, and dogs). Human PK prediction was performed using parameters derived from simultaneous and individual fitting approaches, following the workflow shown in Supplementary Figure S1. The simultaneous fitting approach using the IV data from three species yielded a single set of distribution kinetic parameters ( $f_{d1}$ , $f_{T1}$ , and $K_{p}$ ), which were then used for human PK prediction. In the case of the individual fitting approach, the mean values were calculated from the estimated distribution kinetic parameters for each species (Table 3) and used for human PK prediction. In both approaches, the estimated species-specific CL values were allometrically scaled to predict the human CL . To quantitatively evaluate the predictive performance of the extrapolated human mPBPK models, the absolute average fold error (AAFE) was calculated using the model-predicted plasma concentrations ( $C_{pred}$ ) and the observed clinical data ( $C_{obs}$ ) using Equation 16:

$$AAFE=10^{\frac{1}{n}\sum \left|\log_{10}\left(\frac{C_{pred}}{C_{obs}}\right)\right|}$$ (16)

where n represents the total number of observations. Data processing and numerical computations were performed using Python (version 3.13.2) with the Pandas (version 2.2.3) and NumPy (version 2.2.6) libraries.

**TABLE 3:** PK parameters of propranolol estimated from individual fitting of IV and oral data in each species to the mPBPK model.

Species ( BW , kg)	Value (CV%)							
	$f_{d1}$	$f_{d2}$ ^a^	$f_{T1}$	$f_{T2}$ ^a^	$K_{p}$	CL (mL/min)	F	$k_{a}$ (min^−1^)
Mouse (0.02)	0.938 (3.38)	0.0625 (50.7)	0.610 (12.8)	0.390 (20.1)	7.53 (15.4)	2.85 (6.78)	0.259 (12.1)	0.0141 (14.8)
Rat (0.25)	0.656 (30.6)	0.345 (58.1)	0.239 (79.3)	0.761 (25.0)	5.83 (13.3)	8.88 (12.0)	0.122 (12.9)	0.0649 (25.0)
Rabbit (2.5)	0.756 (15.3)	0.244 (47.4)	0.334 (46.8)	0.666 (23.5)	4.43 (14.4)	156 (7.22)	0.0100 (21.3)	0.00701 (30.0)
Cat (5)	0.870 (7.28)	0.130 (48.5)	0.476 (23.9)	0.524 (21.7)	1.46 (10.3)	129 (5.86)	0.518 (10.3)	0.00927 (10.6)
Dog (10)	0.780 (6.11)	0.221 (21.6)	0.295 (22.0)	0.705 (9.20)	3.39 (6.83)	144 (4.65)	0.0405 (5.83)	0.0191 (13.5)
Human (70)	0.878 (1.90)	0.122 (13.7)	0.237 (12.4)	0.763 (3.85)	6.82 (12.6)	658 (6.32)	0.217 (5.08)	0.00652 (8.54)
Horse (530)	0.915 (2.28)	0.0855 (24.3)	0.526 (10.8)	0.474 (12.0)	1.90 (5.86)	9,330 (3.84)	0.257 (5.69)	0.0204 (10.8)

Model-predicted PK profiles are shown in Figure 4.

^a^ Determined as secondary parameters based on the relationships of $f_{d2}=1-f_{d1}$ and $f_{T2}=1-f_{T1}$ .

## 3 Results

### 3.1 Literature search and data collection

The systematic literature search identified 221 publications, of which 75 contained extractable PK data (Supplementary Table S1). The datasets were further screened, based on sampling density and analytical reliability, resulting in 19 representative datasets (Table 1). These datasets, spanning seven species (mouse, rat, rabbit, cat, dog, horse, and human), were used for allometric scaling and mPBPK modeling to investigate interspecies PK characteristics of propranolol.

### 3.2 Allometric scaling

**FIGURE 2:**
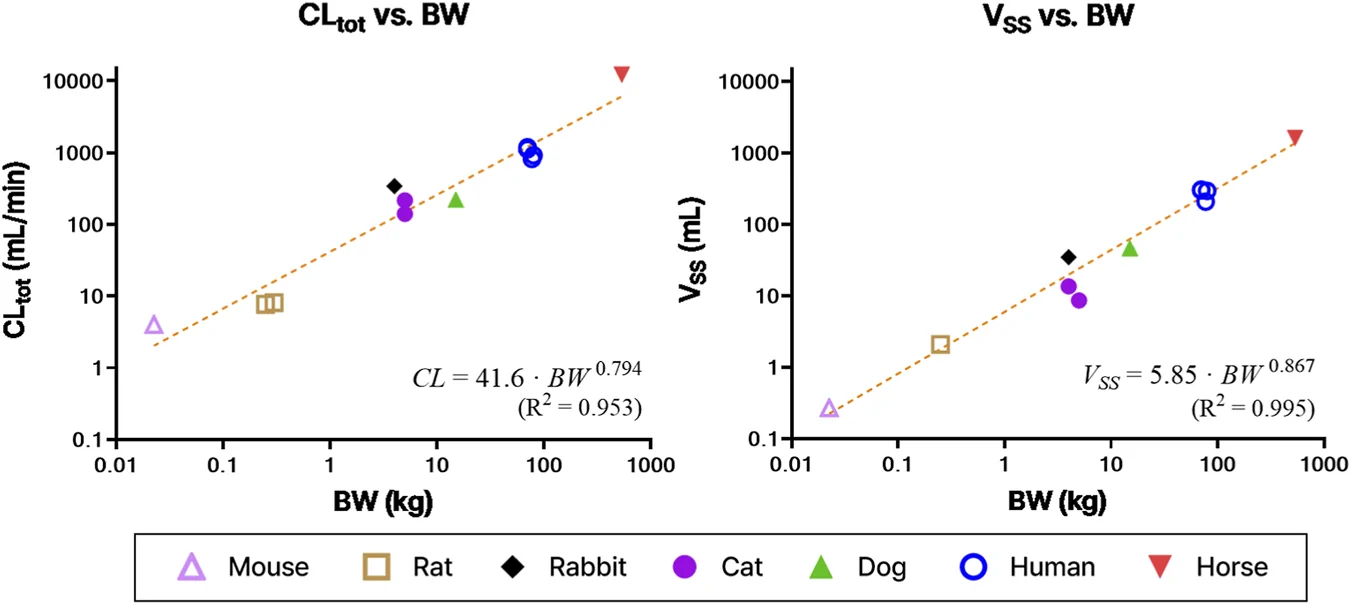
Allometric scaling of $CL_{tot}$ and $V_{SS}$ for propranolol after IV dosing in seven species. The symbols represent individual values reported in the literature or calculated from digitized data (as listed in Table 1). The dashed lines represent the best-fit regression results, with their equations included.

Both $CL_{tot}$ and $V_{SS}$ exhibited robust log-log correlations with the following equations: $CL_{tot}=41.6\cdot BW^{0.794}$ (R^2^ = 0.953) and $V_{ss}=5.85\cdot BW^{0.867}$ (R^2^ = 0.995) (Figure 2). The exponent for $CL_{tot}$ (0.794) was higher than the previously reported value (0.660) (Espie et al., 2009). Deviations from the regression line were observed in several species, with mice (1.97-fold), rabbits (2.73-fold), and horses (2.00-fold) showing higher $CL_{tot}$ values, whereas rats showed a lower value (0.505-fold). With respect to the $V_{SS}$ , deviations were observed for cats (0.357-fold). The $CL_{tot}$ values closely aligned with the $Q_{H}$ values (Supplementary Figure S4B), supporting the notion that hepatic blood flow is a primary determinant of propranolol clearance across species (Jones et al., 1985). The similar values between $Q_{CO}\cdot R_{B}$ and $CL_{D}$ across species support the assumption of perfusion-limited distribution (Supplementary Figure S4C, Supplementary Table S5).

### 3.3 Simultaneous fitting of the IV PK data to the mPBPK model

**FIGURE 3:**
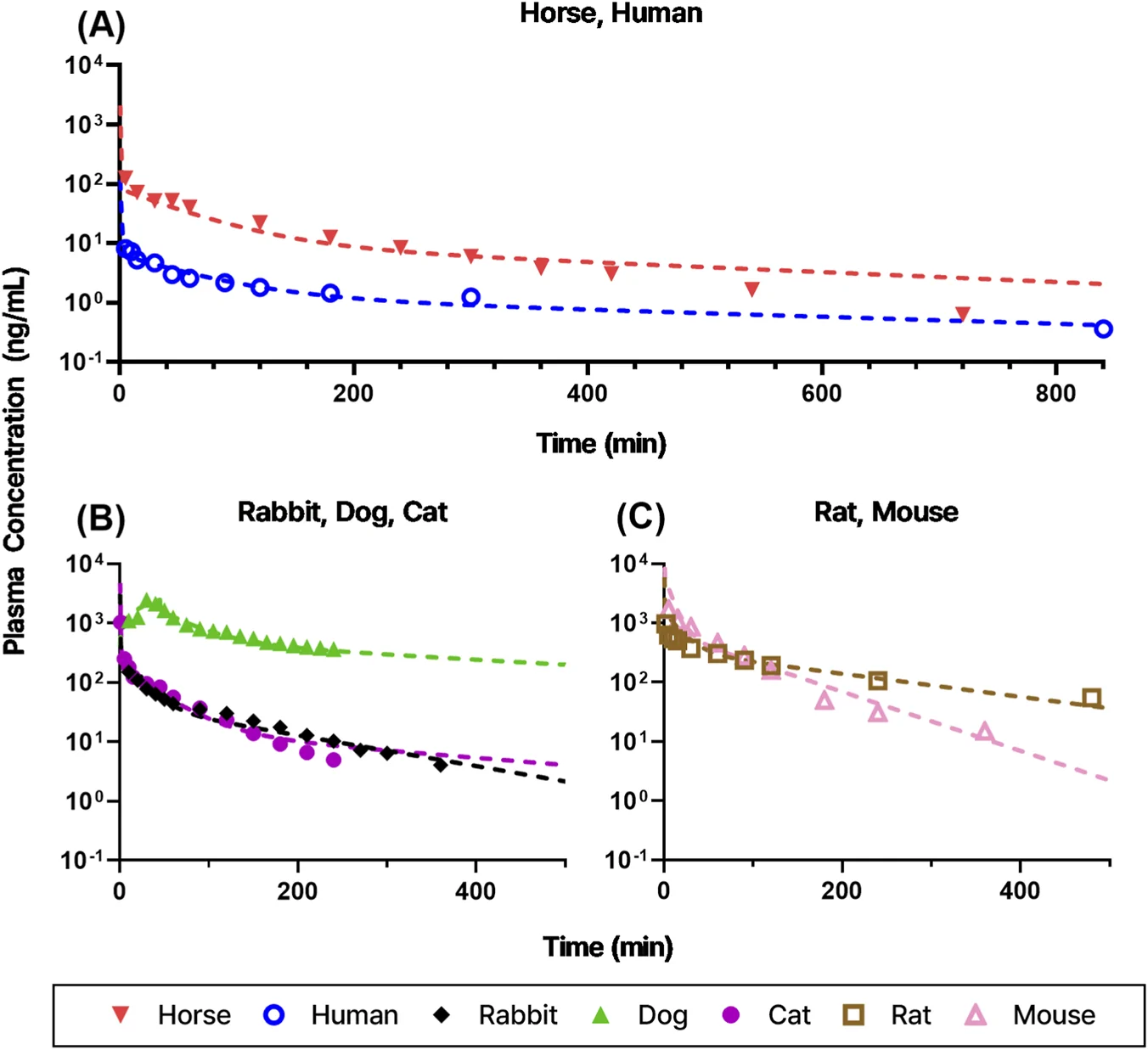
mPBPK model-predicted PK profiles from simultaneous fitting of plasma propranolol PK data after IV dosing in seven species: **(A)** horse and human, **(B)** rabbit, dog, and cat, **(C)** rat and mouse. The symbols represent observed data from the literature (as listed in Table 1), and the dotted lines represent the model-predicted profiles. The estimated parameter values are listed in Tables 2 and 3.

Simultaneous fitting of the IV PK data from seven species to the mPBPK model adequately described the observed PK data (Figure 3). The primary estimated parameters ( $K_{p}$ , $f_{d1}$ , $f_{T1}$ , and CL ) and the secondary parameters ( $f_{d2}$ and $f_{T2}$ ) showed acceptable precision (CV% = 2.02–9.36%; Table 2). The estimated values of $f_{d1}$ and $f_{d2}$ (CV% in parentheses) were 0.820 (2.02%) and 0.180 (9.21%), indicating that most of the cardiac output ( $Q_{CO}$ ) was directed to Tissue 1 relative to Tissue 2. The $K_{p}$ value was estimated to be 4.66 (CV% = 6.45), and species-specific CL values generally agreed with the NCA-derived values.

**TABLE 2:** PK parameters of propranolol estimated from simultaneous fitting of the representative IV data in seven species to the mPBPK model.

Parameter (definition)	Value (CV%)		
Estimated as shared parameters across seven species			
$K_{p}$ (Tissue-to-plasma partition coefficient)	4.66 (6.45)		
$f_{d1}$ (Fractional distribution parameter for Tissue 1)	0.820 (2.02)		
$f_{d2}$ ^a^ (Fractional distribution parameter for Tissue 2)	0.180 (9.21)		
$f_{T1}$ (Fraction of total tissue volume for Tissue 1)	0.232 (9.36)		
$f_{T2}$ ^a^ (Fraction of total tissue volume for Tissue 2)	0.768 (2.83)		
*CL estimated for each species*	mL/min	mL/min/kg	CV%
Mouse	2.20	110	5.54
Rat	6.92	27.7	7.96
Rabbit	145	58.1	4.44
Cat	168	33.5	5.75
Dog	120	12.0	9.83
Human	599	8.55	11.6
Horse	9,150	17.3	5.88

Model-predicted PK profiles are shown in Figure 3.

^a^ Determined as secondary parameters based on the relationships of $f_{d2}=1-f_{d1}$ and $f_{T2}=1-f_{T1}$ .

### 3.4 Individual fitting of IV and oral data for each species

**FIGURE 4:**
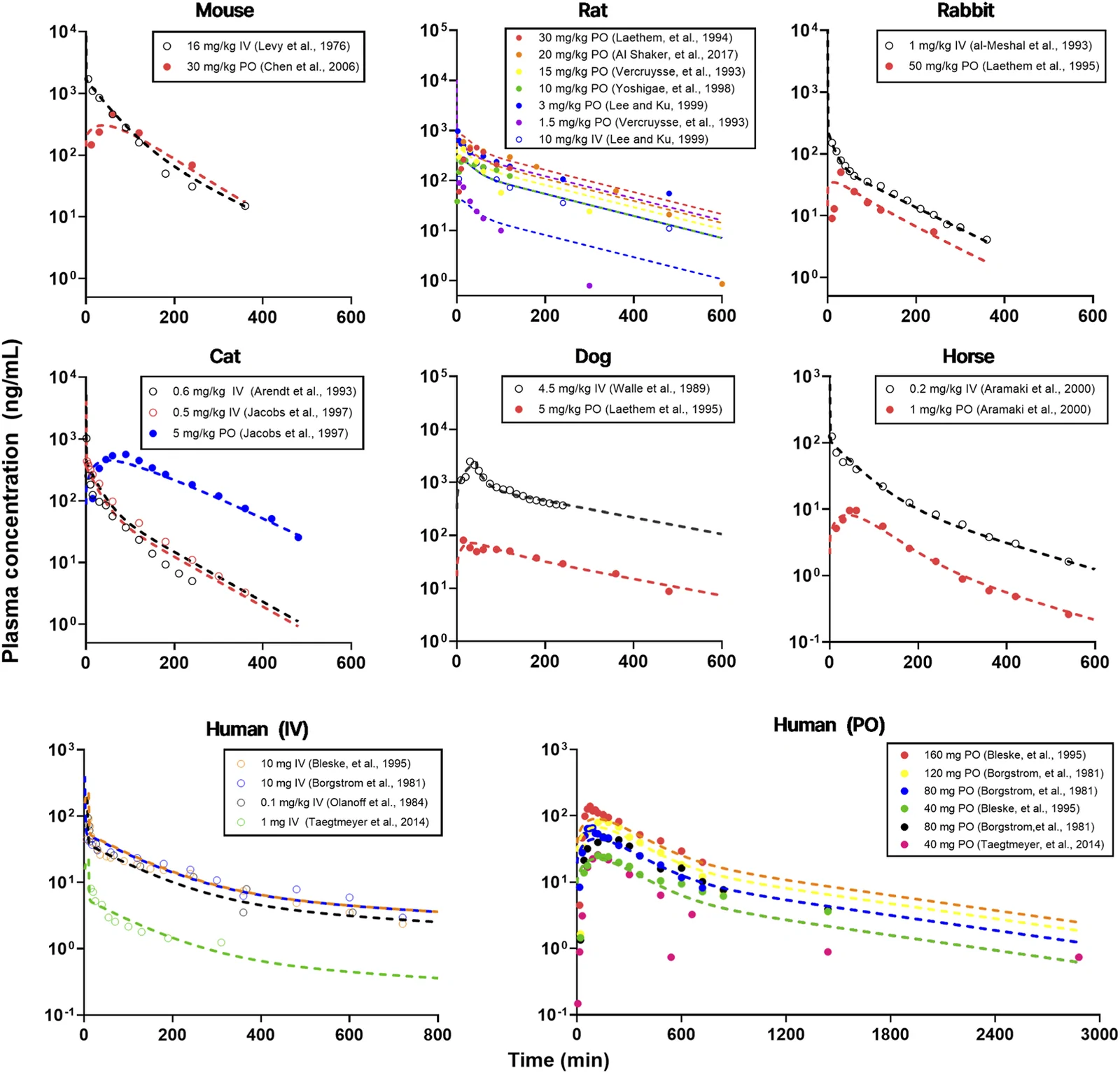
mPBPK model-predicted PK profiles from individual fitting of plasma propranolol PK data after IV and oral dosing in each species (mouse, rat, rabbit, cat, dog, horse, and human). The symbols denote the observed data, and the dotted lines denote the model-predicted profiles. Corresponding literature sources and dosage information are presented in colors. The estimated parameters are listed in Table 3.

When the IV and oral data from each species were individually fitted to the mPBPK model, the model-predicted PK profiles were in good agreement with the observed data (Figure 4), and most parameter estimates were obtained with acceptable precision ranging from 1.90% to 79.3% (Table 3). The distribution kinetic parameters ranged from 0.656 to 0.938 ( $f_{d1}$ ), from 0.0625 to 0.345 ( $f_{d2}$ ), and from 0.237 to 0.610 ( $f_{T1}$ ) across species. Oral absorption kinetic parameters showed variability, with F ranging from 0.0100 to 0.518 and $k_{a}$ ranging from 0.00652 to 0.0649 min^−1^. The parameters F and $F\cdot k_{a}$ showed no clear relationship with BW , while a weak correlation was observed for $k_{a}$ ( $k_{a}=0.0161\cdot BW^{-0.07}$ , R^2^ = 0.106), as presented in Supplementary Figure S5.

### 3.5 The unitless product-ratio plot for the biological half-lives of propranolol across species

**FIGURE 5:**
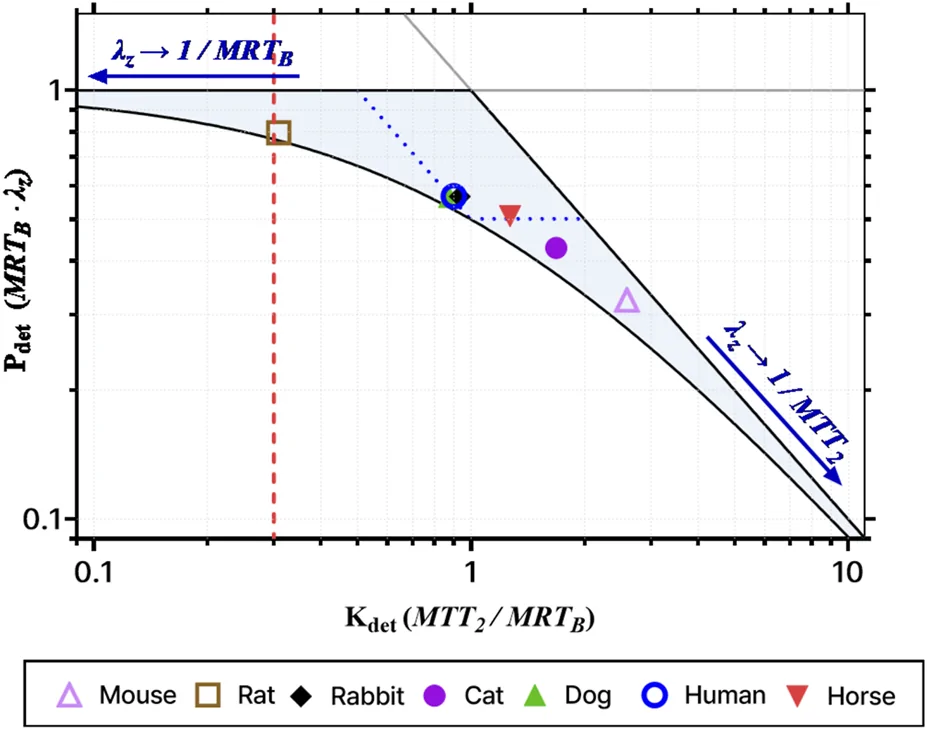
Unitless product-ratio plot ( $P_{\det}$ versus $K_{\det}$ ) evaluating the terminal-phase half-lives of propranolol across seven species. The dimensionless parameters were derived from individual mPBPK model fittings. The light blue-shaded region enclosed by the solid lines denotes the theoretical space of the terminal half-life, and the dotted lines indicate the threshold conditions where $P_{\det}=0.5$ and $P_{\det}\cdot K_{\det}=0.5$ . The vertical red dashed line marks the condition where $K_{\det}=0.3$ .

The unitless product-ratio plot was applied to the mPBPK model fitting results to determine the limiting condition of $T_{1/2}$ across species (Figure 5). The product $P_{\det}=$ $MRT_{B}{\cdot \lambda }_{z}$ and the ratio $K_{\det}=MTT_{2}/MRT_{B}$ were calculated from the individually fitted parameters (Table 3) using Equations 12–14, as listed in Table 4. Based on the theoretical threshold of 0.5 (two dotted lines within the light blue-shaded space in Figure 5), rats, dogs, and humans showed $P_{\det}>0.5$ and $P_{\det}K_{\det}<0.5$ , indicating that the biological half-life of propranolol in these species is more closely associated with $MRT_{B}$ . In contrast, mice and cats showed $P_{\det}<0.5$ and $P_{\det}K_{\det}>0.5$ , indicating that $T_{1/2}$ is more closely associated with $MTT_{2}$ . For rabbits and horses, both $P_{\det}$ and $P_{\det}K_{\det}$ were greater than 0.5, indicating that $T_{1/2}$ is kinetically associated with both $MRT_{B}$ and $MTT_{2}$ . When the previously proposed $K_{\det}$ threshold of 0.3 for discrimination of the number of tissue compartments in the rat mPBPK models (Jeong et al., 2022a, b) was applied across species, all species showed $K_{\det}>0.3$ (left red vertical dashed line in Figure 5). These results support the applicability of the two-tissue mPBPK structure for the description of propranolol PK across all species examined.

**TABLE 4:** Summary of the time constants and slopes used for calculations of the unitless products ( $P_{\det}$ , $K_{\det}$ , and $P_{\det}\cdot K_{\det}$ ).

Species	$MRT_{B}$ (min)	$\lambda_{z}$ (min^−1^)	$MTT_{1}$ (min)	$MTT_{2}$ (min)	$P_{\det}$	$K_{\det}$	$P_{\det}\cdot K_{\det}$
Mouse	48.8	0.00664	13.2	126	0.324	2.59	0.840
Rat	156	0.00509	8.00	48.4	0.796	0.309	0.246
Rabbit	67.2	0.00842	10.2	62.7	0.566	0.933	0.528
Cat	74.1	0.00570	12.3	127	0.423	1.72	0.726
Dog	142	0.00398	4.11	124	0.564	0.875	0.494
Human	678	0.000847	25.0	582	0.575	0.857	0.493
Horse	94.7	0.00537	19.7	120	0.509	1.27	0.646

The values were obtained from the individual mPBPK model fitting for each species, and plotted on the unitless-product ratio plot (Figure 5).

### 3.6 Identification of tissue constituents in the mPBPK model for propranolol in mice, rats, and human

To identify the anatomical tissue constituents corresponding to the Tissue 1 and Tissue 2 compartments, the WB-PBPK models were constructed for mice, rats, and humans using the CL estimates obtained from the individual fitting (Table 3) and physiological information (Supplementary Table S3). The experimentally determined $K_{p}$ values were adopted for rats (Supplementary Equation S1), while the $K_{p}$ values were further refined for mice (Supplementary Equation S9) and humans (Supplementary Table S10), as summarized in Supplementary Table S4. The resulting WB-PBPK models reasonably captured the observed IV PK data (gray solid lines, Supplementary Figure S6), with AAFE values of 1.26 (mice), 1.50 (rats), and 1.14 (humans).

The reduced model generated by progressive tissue lumping yielded plasma PK profiles that nearly overlapped with those from the WB-PBPK models (blue dashed lines and gray solid lines, Supplementary Figure S6). In the reduced model, the SEG was identified as primarily consisting of brain and muscle in mice; skin, muscle, and brain in rats; and bone, muscle, and adipose tissue in humans (Table 5). The apparent tissue volumes ( $\sum V_{T,i}{\cdot K}_{p,i}$ ) and distributional clearances ( $\sum Q_{T,i}\cdot R_{B}$ ) estimated from the progressive tissue lumping were quantitatively consistent with the corresponding mPBPK parameter estimates for both the SEG and REG across mice, rats, and humans. For the SEG, the bottom-up estimates of $\sum V_{T,i}{\cdot K}_{p,i}$ and $\sum Q_{T,i}\cdot R_{B}$ were 60.2 mL and 1.31 mL/min in mice, 603 mL and 22.4 mL/min in rats, and 238 L and 1,480 mL/min in humans, which were comparable to the corresponding top-down estimates ( $V_{2}\cdot K_{p}$ and $Q_{CO}{\cdot f}_{d2}\cdot R_{B}$ ) of 53.7 mL and 0.424 mL/min in mice, 1,060 mL and 21.7 mL/min in rats, and 337 L and 580 mL/min in humans. The same applies to the REG (Table 5). Overall, these findings support the physiological relevance of the mPBPK structure consisting of the two tissue compartments (Tissues 1 and 2) and its ability to capture the major distributional characteristics across species.

**TABLE 5:** Physiological and drug-specific parameters ( $K_{p}$ and MTT ) used in WB-PBPK models for propranolol in mice, rats, and humans, compared to the relevant distribution-related parameters from the reduced model (including the REG and SEG compartments) and the mPBPK model (including Tissues 1 and 2 compartments).

​	Mouse					Rat					Human				
	Tissue	$V_{T}$ (mL)	$Q_{T}$ (mL/min)	$K_{p}$ ^a^	MTT ^b^ (min)	Tissue	$V_{T}$ (mL)	$Q_{T}$ (mL/min)	$K_{p}$ ^a^	MTT ^b^ (min)	Tissue	$V_{T}$ (mL)	$Q_{T}$ (mL/min)	$K_{p}$ ^a^	MTT ^b^ (min)
WB-PBPK model	Muscle	7.68	1.27	6.76	48.0	Skin	47.6	4.29	2.54	33.1	Bone	10,000	235	4.35	218
	Brain	0.330	0.264	25.0	36.8	Muscle	101	20.6	4.53	26.2	Muscle	28,000	1,220	6.02	163
	Skin	3.31	0.464	3.79	31.7	Brain	1.43	1.48	16.8	19.0	Adipose	15,000	291	1.69	102
	SPL	1.94	1.29	11.1	19.7	SPL	15.9	13.5	7.35	10.2	Brain	1,400	638	22.3	57.6
	Liver^c^	1.10	1.29	5.61	5.63	Liver^c^	9.15	13.5	3.76	2.99	Skin	2,600	336	3.37	30.7
	Gut^c^	0.844	0.336	18.2	53.8	Gut^c^	6.75	7.50	12.2	12.9	SPL	3,000	1,270	9.48	26.3
	Bone	2.15	0.880	4.88	14.0	Bone	18.2	9.03	3.27	7.78	Liver^c^	1,800	1,270	5.00	8.33
	Kidney	0.334	0.728	24.5	13.2	Kidney	1.83	10.4	16.5	3.39	Gut^c^	1,200	1,100	16.2	20.8
	Adipose	1.90^d^	0.560	1.89	7.55	Heart	0.825	3.77	8.05	2.07	Heart	329	224	10.7	18.5
	Heart	0.100	0.528	12.0	2.67	Adipose	6.64	5.18	1.27	1.91	Kidney	308	980	21.9	8.08
Reduced model	$\sum V_{T,i}\cdot K_{p,i}$ (mL)			$\sum Q_{T,i}\cdot R_{B}$ (mL/min)			$\sum V_{T,i}\cdot K_{p,i}$ (mL)		$\sum Q_{T,i}\cdot R_{B}$ (mL/min)			$\sum V_{T,i}\cdot K_{p,i}$ (mL)		$\sum Q_{T,i}\cdot R_{B}$ (mL/min)	
	SEG	60.2		1.31		SEG	603		22.4		SEG	238,000		1,480	
	REG	57.5		3.78		REG	222		35.7		REG	78,700		2,930	
mPBPK model^e^	$V_{i}\cdot K_{p}$ (mL)			$Q_{CO}f_{di}\cdot R_{B}$ (mL/min)		$V_{i}\cdot K_{p}$ (mL)			$Q_{CO}f_{di}\cdot R_{B}$ (mL/min)			$V_{i}K_{p}$ (mL)		$Q_{CO}f_{di}\cdot R_{B}$ (mL/min)	
	Tissue 2	53.7		0.424		Tissue 2	1,060		21.7		Tissue 2	337,000		580	
	Tissue 1	84.2		6.38		Tissue 1	266		41.2		Tissue 1	105,000		4,180	

The shaded region represents the organs identified as the SEG.

^a^ $K_{p}$ is equivalent to the tissue-to-plasma partition coefficient ( $K_{p,SS}$ ): Supplementary Equation S1 for rats, Supplementary Equation S9 for mice, and Supplementary Equation S10 for humans.

^b^ Calculated using Equation 15.

^c^ Constituents of the SPL, compartment.

^d^ The fraction of the cardiac output to the tissue was assumed identical between rats and mice (Brown et al., 1997).

^e^ The mPBPK, model parameters were derived from Table 3.

### 3.7 Prediction of human PK profiles after IV dosing

**FIGURE 6:**
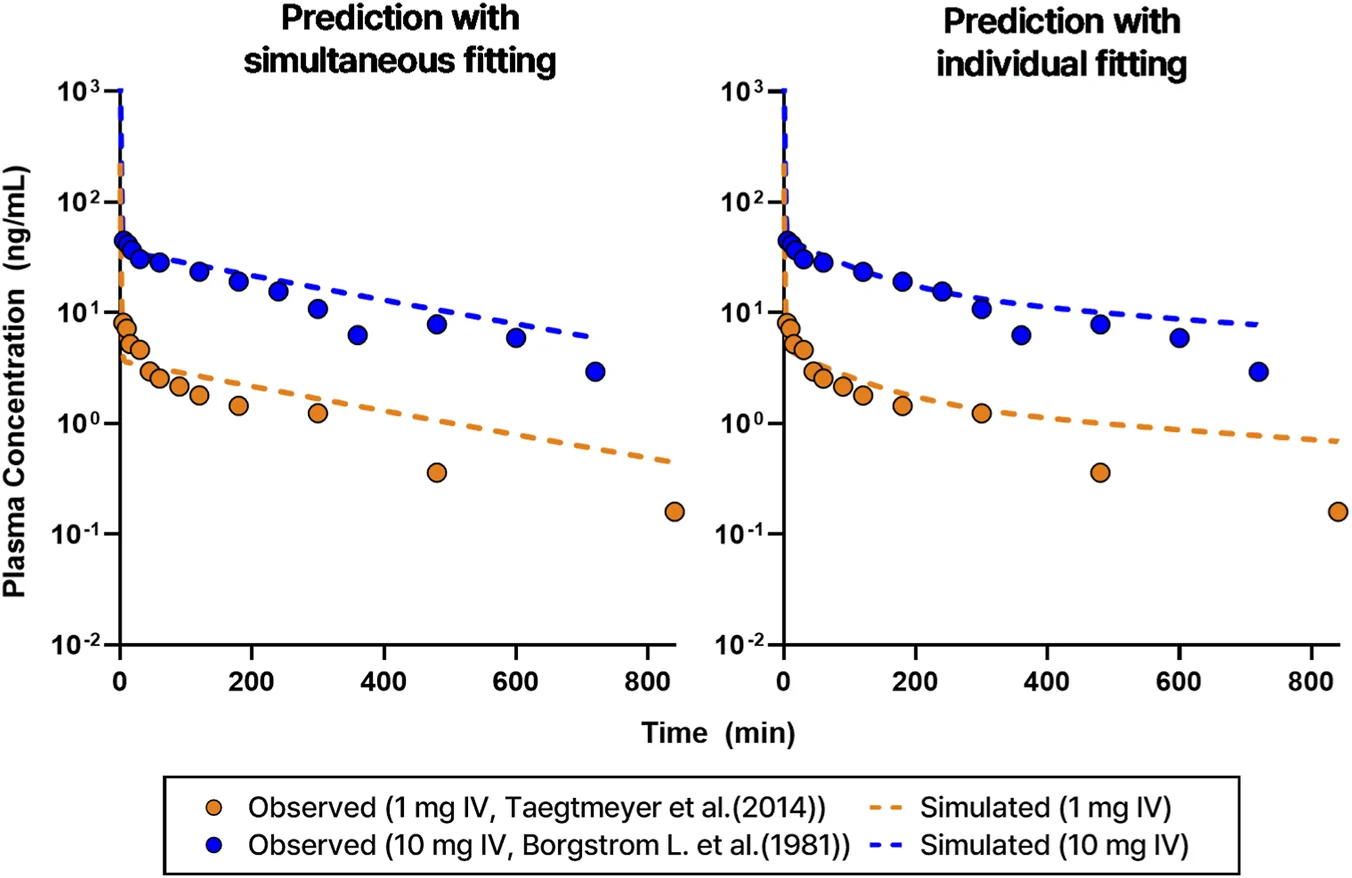
Simulated plasma PK profiles of propranolol in humans after IV dosing (1 or 10 mg) using predicted mPBPK parameters. The left panel shows the prediction using parameters derived from the simultaneous fitting of the three preclinical species (mouse, rat, and dog). The right panel shows the prediction using the mean values of the distribution kinetic parameters estimated from the individual fitting. In both approaches, the human systemic clearance was extrapolated from the fitted preclinical values via allometric scaling.

We predicted the human PK profiles of propranolol following IV administration using the mPBPK model parameters derived from three preclinical species (mice, rats, and dogs) and the human physiological parameters (Supplementary Table S2). Both the simultaneous and individual fitting approaches adequately reproduced the observed human PK profiles at 1 and 10 mg IV doses (Figure 6). The individual-fitting approach yielded AAFE values of 1.35 (1 mg) and 1.28 (10 mg), while the simultaneous-fitting approach resulted in comparable AAFE values of 1.45 (1 mg) and 1.33 (10 mg).

## 4 Discussion

The current study curated a rich body of propranolol PK data across multiple mammalian species, and established a unified cross-species mPBPK model. Prediction of drug disposition kinetics in humans is important for clinical drug development as human IV PK data are often unavailable in clinical settings. The current study demonstrates the utility of the interspecies modeling approach for predicting the human IV PK profiles of propranolol (Figure 6) by incorporating the distribution kinetic parameters shared across species in the mPBPK modeling framework. The comparison between top-down mPBPK and bottom-up WB-PBPK models improved physiological interpretation of the peripheral compartments by identifying the corresponding anatomical tissues in mice, rats, and humans (Table 5). Application of the unitless product-ratio plot to the interspecies PK analysis results provided a mechanistic interpretation of the substantial cross-species variability in $T_{1/2}$ , informing whether $T_{1/2}$ was governed by the $MRT_{B}$ -limited or $MTT_{\max}$ -limited condition (Figure 5).

Conventional allometric scaling of $CL_{tot}$ and $V_{SS}$ displayed log-log linear relationships with BW , with the respective exponents of 0.794 and 0.867 (Figure 2). The allometric relationship for $CL_{tot}$ closely aligned with that for $Q_{H}$ (Supplementary Figure S4), suggesting that $Q_{H}$ is a major physiological determinant of propranolol clearance not only in rats (Jones et al., 1985) but also in other mammalian species. Although the exponent of 0.867 for $V_{SS}$ deviated from unity, the assumption of the shared distribution kinetic parameters appeared generally valid given the reasonable performance of the simultaneous mPBPK modeling across the seven species (Table 2; Figure 3). The extent of tissue distribution of propranolol was reported to be governed by plasma protein binding (Branch et al., 1976). Due to the lack of information on the plasma unbound fraction ( $f_{u,p}$ ) for most species, species differences in plasma protein binding were not incorporated into the current mPBPK model. However, when the WB-PBPK models incorporated the species-dependent $f_{u,p}$ values [e.g., 0.032 for mice assuming the same value as rats (Shibasaki et al., 1989), 0.114 for humans (Taylor and Turner, 1981)], the scaling factors for the $K_{p}$ values were 1.49 [CV% = 15.7%; Supplementary Equation S9] for mice and 0.373 [13.5%; Supplementary Equation S10] for humans and yielded acceptable model performance (Supplementary Figure S6). Overall, these findings suggest that propranolol displays tissue distribution characteristics that are largely conserved across species, and that accounting for species-dependent plasma protein binding can explain some of the observed interspecies variability in $V_{SS}$ .

The current analyses were conducted under the assumption that the rate of tissue distribution for propranolol is perfusion-limited. To examine the validity of this assumption, the total distributional clearance ( $CL_{D}$ ) was calculated using the intercepts and slopes obtained from fitting the plasma PK data to tri-exponential functions: $CL_{D}=Dose\cdot \left(\sum \left(C_{i}\cdot \lambda_{i}\right)/C_{0}^{2}-1/AUC\right)$ (Supplementary Table S5) (Veng-Pedersen, 1984; Gillespie and Veng-Pedersen, 1985). The initial concentration ( $C_{0}$ ) was constrained to $Dose/\left(V_{B}{\cdot R}_{B}\right)$ to facilitate direct comparison between the calculated $CL_{D}$ and the apparent plasma flow $Q_{CO}{\cdot R}_{B}$ . The calculated $CL_{D}$ values were comparable with the corresponding $Q_{CO}\cdot R_{B}$ values across species, supporting that the rate of propranolol distribution to tissues is predominantly perfusion-limited (Supplementary Figure S4C). An *in vitro* apparent permeability coefficient ( $=f_{u,p}\cdot P/R_{B}$ ) of 0.88 × 10^−6^ cm/s (Jeong et al., 2017) further supported this interpretation, yielding an estimated $\sum \left(Q_{T}\cdot f_{d}\right)$ value of 63.8 mL/min, which was comparable with $Q_{CO}$ in rats (74 mL/min). In addition, given the relationship $ER<f_{d}<1$ in eliminating organs (Jeong and Jusko, 2022a; 2024), the high hepatic extraction of propranolol ( ER≈1 ) implies that membrane permeability is unlikely to be rate-limiting in the liver ( $f_{d}\approx 1$ ). Collectively, these findings validate the assumption of perfusion-limited distribution for propranolol. Accordingly, $f_{d1}$ was determined as one of the primary estimated parameters, while $f_{d2}$ was derived secondarily via the relationship $f_{d1}+f_{d2}=1$ .

Given that the mPBPK model is typically constructed using plasma data alone, the peripheral compartments are not explicitly resolved in terms of the anatomical tissues. Jeong et al. (2022a) and Jeong et al. (2022b) proposed a progressive tissue lumping approach, in which tissues are sequentially aggregated from the tissue with the longest MTT . In that study, based on the analysis of 113 model compounds, a fractional change in the terminal slope ( FCT ) < 10% following tissue lumping was proposed as a practical threshold for preserving the PBPK model calculations. For propranolol, a more conservative criterion appeared necessary to reconcile the WB-PBPK and mPBPK model structures (e.g., FCT < 1%). Assuming that the constructed WB-PBPK models adequately represented tissue disposition (Supplementary Figure S6), the SEG was identified as consisting of muscle and brain in mice; skin, muscle, and brain in rats; and bone, muscle, and adipose tissue in humans (Table 5). These species-dependent compositions reflect differences in tissue volume, blood-flow allocation, and tissue partitioning, which jointly determine tissue transit kinetics and the contribution of each tissue to the terminal disposition of the overall system. Of note, mouse skin represented a borderline tissue because its MTT was similar to that of brain, and its inclusion in the SEG produced nearly indistinguishable PBPK simulations. Accordingly, its assignment to the REG reflects the applied lumping criterion rather than a clear kinetic separation from brain. Overall, these findings indicate that the anatomical composition of the lumped compartments may differ across species, even when the same mPBPK model structure is applied. Of note, applying the rat $f_{u,p}$ value to the human WB-PBPK model yielded the same SEG composition as that obtained using the human $f_{u,p}$ value (i.e., bone, muscle, and adipose tissue). Although plasma protein binding affects the overall extent of tissue distribution, it does not appear to fully explain the species-dependent SEG and REG compositions under the present model assumptions.

We performed a local sensitivity analysis by varying $f_{T1}$ , $f_{d1}$ , $K_{p}$ , and CL around their top-down estimates and quantifying the deviation from the observed IV plasma concentration-time profiles (Supplementary Figure S7). While the profiles were sensitive to changes in each parameter, the bottom-up estimates were within two-fold of their corresponding top-down estimates and generally located in regions of relatively small deviations from the observed profiles across the species. These results indirectly support the kinetic relevance of the proposed tissue assignments. A more direct validation approach would involve comparison with experimental tissue concentration-time data particularly in mice and humans.

To determine the limiting condition of the terminal-phase $T_{1/2}$ of propranolol across species, the unitless product-ratio plot was constructed by plotting $P_{\det}$ ( $MRT_{B}\cdot \lambda_{z}$ ) versus $K_{\det}$ ( $MTT_{\max}/MRT_{B}$ ) (Figure 5). A key feature of the unitless product-ratio plot is that $\lambda_{z}$ , which is obtained as a model-independent parameter from the NCA of the observed data, is confined to a defined theoretical space (Equation 9) regardless of the number of peripheral compartments in PBPK systems. This theoretical dimensional reduction can differentiate whether $\lambda_{z}$ is primarily governed by $1/MRT_{B}$ (i.e., $P_{\det}>0.5$ ) or by $1/MTT_{\max}$ (i.e., $P_{\det}\cdot K_{\det}>0.5$ ). Based on our interspecies analysis (Figure 5), rats and humans showed the $P_{\det\;}$ values of 0.796 and 0.575, indicating that the terminal phase is governed by the overall disposition kinetics ( $\lambda_{z}∼1/MRT_{B}$ ). The $P_{\det}$ value of 0.324 and the $P_{\det}\cdot K_{\det}$ value of 0.840 (Table 4) in mice indicate that the terminal phase is governed by the transit kinetics through the tissues in the SEG ( $\lambda_{z}∼1/MTT_{\max}$ ), which were identified to be muscle and brain (Table 5).

In terms of oral absorption kinetics, the current findings from the interspecies PK analysis of propranolol differ from those reported previously for metformin (Jeong and Jusko, 2021). Metformin demonstrated a negative correlation of F and $F\cdot k_{a}$ against BW , whereas the current analysis for propranolol did not (Supplementary Figure S5B). Propranolol exhibits high intestinal permeability, and its fraction absorbed from the intestinal lumen ( $F_{a}$ ) is expected to be nearly complete across species (Yu and Amidon, 1999; Agoram et al., 2001), which is consistent with our ACAT (advanced compartmental absorption and transit) model calculations (e.g., $F_{a}$ ≥ 89.7% for all species) (Yu and Amidon, 1999; Jeong and Jusko, 2021). However, the mPBPK analysis revealed relatively low and variable F values (e.g., 1.00% in rabbits, 4.07% in dogs, and 48.1% in cats). Within the well-stirred model (Rowland et al., 1973), such behavior is consistent with the extensive hepatic extraction (i.e., $f_{u,p}\cdot CL_{u,\mathrm{int}}\gg Q_{H}$ ) where the hepatic availability is approximated as $F_{h}\approx Q_{H}/\left(f_{u,p}\cdot CL_{u,\mathrm{int}}\right)$ . Accordingly, the lack of a clear relationship between F and BW (Supplementary Figure S5B) is unlikely to reflect differences in the intestinal absorption process, but rather species-dependent variability in the hepatic extraction. We attempted to predict the oral PK profiles in humans using the extrapolated parameters including F and $k_{a}$ from the three preclinical species, but the predictive performance was poor (Supplementary Figure S8) unlike IV predictions (Figure 6). The poor predictive performance of oral PK likely stems from pronounced species dependence in hepatic extraction. The similarity between $k_{a}$ and $2\cdot P_{eff}/R$ across species, where $P_{eff}$ is the effective intestinal permeability and R is the gut radius (Supplementary Figure S5C), suggests that the intestinal permeation may primarily govern the rate process of its oral absorption in the species assessed and captured by our interspecies modeling effort.

Several limitations of the present study warrant further consideration. First, the present analysis was conducted under the assumption of linear PK. However, previous literature has suggested the possibility of non-linear PK, albeit within a limited dose range (Komura et al., 2005). Given the substantial inter-study variability identified in our literature review (Supplementary Figure S2), future investigations spanning a wider dose range would be necessary to verify dose proportionality. Second, this study primarily focused on the PK analysis of propranolol as a racemate. It is well-recognized that the absorption, distribution (Walle et al., 1989; Vermeulen et al., 1992), and elimination (Olanoff et al., 1984) of propranolol may be stereoselective. As summarized in the compiled literature (Supplementary Table S1), such stereoselective PK behaviors vary across species and experimental settings. In addition, the present analysis did not fully resolve strain-level dependence in the compiled PK data, which may have contributed to the remaining inter-study variability.

## 5 Conclusion

In this study, a top-down, stepwise mPBPK modeling approach quantitatively characterized the absorption and disposition kinetics of propranolol across seven mammalian species. By integrating top-down PK analysis with bottom-up physiological information, the proposed framework enabled mechanistic interpretation of the mPBPK structure through reconciliation with tissue-specific distribution kinetics. The unitless product-ratio plot provided a quantitative means to determine whether the terminal-phase kinetics were primarily governed by 1/ $MRT_{B}$ or 1/ $MTT_{\max}$ , revealing the species-dependent limiting conditions of propranolol $T_{1/2}$ . The current mPBPK framework also demonstrated acceptable translational performance, predicting human IV PK profiles from preclinical data. Collectively, these findings illustrate how mechanistically informed mPBPK analysis can facilitate physiological interpretation of interspecies PK behavior and quantitative preclinical-to-clinical translation.

## Data Availability

The original contributions presented in the study are included in the article/Supplementary Material, further inquiries can be directed to the corresponding authors.
