# Supplementary material for: Multi-species assessment of propranolol pharmacokinetics via minimal and whole-body PBPK modeling
Source: Front Pharmacol. 2026 Sep 10;17:1933766. doi: 10.3389/fphar.2026.1933766 (PMC13601250; doi:10.3389/fphar.2026.1933766)
Supplement: Supplementary file 1 [file DataSheet1.pdf]

Supplementary Material

1 Method

1.1 Development of the WB-PBPK model for propranolol PK in rats and humans

The WB-PBPK model consisted of the blood and nine tissue compartments, i.e., skin (SK), muscle (MU), adipose (AD), bone (BO), brain (BR), heart (HE), kidney (KI), lung (LU), and splanchnic (SPL) compartment, as shown in **Supplementary Fig. S3**. The physiological parameters used for construction of the model are listed in **Supplementary Table S3**. Tissues for which the distribution data were not available (e.g., adrenal glands, pancreas, and spleen) or the minimal contribution was expected in terms of Q_T and V_T (e.g., testes and thyroid) were excluded from model construction. For a fair comparison between the WB-PBPK and mPBPK models, some structural modifications were considered in development of the WB-PBPK model, by assuming (i) elimination occurring from plasma (i.e., CL), (ii) the venous and arterial blood pooled into a single compartment (V_B), and (iii) splanchnic tissues (i.e., gut and liver) lumped into the SPL compartment such that the 9 peripheral compartments are connected in parallel to the blood. Given the high lipophilicity (log P = 3.48) and apparent permeability coefficient ($f_{up}P/R_B$ = 0.88 × 10^{-6} cm/s) of propranolol (Jeong et al., 2017), perfusion-limited model was assumed for its tissue distribution kinetics, consistent with the previous ones (Poulin and Theil, 2002; Cheung et al., 2018).

The stereoselective disposition of propranolol for major tissues was reported in the literature (Rodgers et al., 2005), with respect to the enantiomer-specific tissue-to-plasma water 'partition coefficients' ($K_{pu,R}$ and $K_{pu,S}$) observed at steady-state after the infusion of the racemate (**Supplementary Table S4**). The steady-state tissue-to-plasma 'concentration ratios' corresponding to the racemate ($K_{p,SS}$) can be expressed as:

$$K_{p,SS} = \frac{C_{T,SS,R}+C_{T,SS,S}}{C_{p,SS,R}+C_{p,SS,S}} = \frac{\frac{C_{T,SS,R}}{C_{p,SS,R}}\cdot\frac{C_{p,SS,R}}{C_{p,SS,S}}+\frac{C_{T,SS,S}}{C_{p,SS,S}}}{\frac{C_{p,SS,R}}{C_{p,SS,S}}+1} \quad \text{(S1)}$$

where $C_{T,SS}$ and $C_{p,SS}$ represent the tissue and plasma concentrations at steady-state. The steady-state tissue-to-plasma concentration ratios for R- and S-propranolol can be calculated using the free fraction in the rat plasma ($f_{up,R} = 0.017$ and $f_{up,S} = 0.127$) (Rodgers et al., 2005), expressed as:

$$\frac{C_{T,SS,R}}{C_{p,SS,R}} = K_{pu,R}\cdot(1-ER)\cdot f_{up,R} \quad \text{(S2)}$$

$$\frac{C_{T,SS,S}}{C_{p,SS,S}} = K_{pu,S}\cdot(1-ER)\cdot f_{up,S} \quad \text{(S3)}$$

where ER is the extraction ratio. Given that propranolol is primarily eliminated by the liver with the negligible renal clearance (Routledge and Shand, 1979; Bargar et al., 1983), the ER was assumed to be zero except for the liver (0.73) (Singh et al., 1991) The plasma exposure ratio between the enantiomers (i.e., $C_{p,SS,R}/C_{p,SS,S}$) in Eq. (S1) was considered to be identical to the AUC ratio following an IV dose of the racemate in rats (i.e., AUC_R/AUC_S), calculated based on their respective plasma

clearance (Vermeulen et al., 1992). The $K_{p,SS}$ values resulting from Eq. (S1) were subsequently utilized in the WB-PBPK model refinement (see below).

Considering the physiological vicinity, the splanchnic tissues, i.e., liver and gut, were lumped into a single SPL compartment, based on the tissue lumping principle (Jeong et al., 2022a; b):

$$V_{SPL} = V_{GU} + V_{LI} \tag{S4}$$

$$Q_{SPL} = Q_{LI} \tag{S5}$$

$$K_{p,SS,SPL} = \frac{V_{GU} \cdot K_{p,SS,GU} + V_{LI} \cdot K_{p,SS,LI}}{V_{GU} + V_{LI}} \tag{S6}$$

where V_{SPL} and Q_{SPL} are the anatomical tissue volume and blood flow for the SPL compartment.

Accordingly, the differential equation for the 9 tissue compartments ($i = 1 \sim 9$) was described in terms of the tissue concentration ($C_{T,i}$) as:

$$V_{T,i} \frac{dC_{T,i}}{dt} = Q_{T,i} \cdot R_B \cdot \left(C_p - \frac{C_{T,i}}{K_{p,i}} \right) \tag{S7}$$

where $V_{T,i}$ is the anatomical tissue volume, $Q_{T,i}$ is the blood flow to the tissue, R_B is the blood-to-plasma partition coefficient, and $K_{p,i}$ is the tissue-to-plasma concentration ratio. The differential equation for the blood compartment is expressed as:

$$V_B \cdot R_B \frac{dC_p}{dt} = -\sum_{i=1}^{9} Q_{T,i} \cdot R_B \cdot \left(C_p - \frac{C_{T,i}}{K_{p,i}} \right) - CL \cdot C_p \tag{S8}$$

The CL values in Eq. (S8) were fixed as the values determined from the individual fitting of the mPBPK model (Table 3).

For rats, the tissue-specific $K_{p,i}$ values were obtained from Eq. (S1) (i.e., $K_{p,i} = K_{p,SS,i}$). For mice and humans, the WB-PBPK model was further optimized using the corresponding plasma PK data, where the scaling factors (SF_{mouse} and SF_{man}) were considered to assess the impact of the potential interspecies differences between the rat $K_{p,SS}$ [Eq. (S1)] and the mouse and human $K_{p,i}$ [Eqs. (S7) and (S8)]. For mice, the $K_{p,i}$ values were expressed as:

$$K_{p,i} = K_{p,SS,i} \cdot SF_{mouse} \tag{S9}$$

Since rats and humans exhibited different f_{up} values of propranolol [e.g., $f_{up,rat}$ = 0.032 (Shibasaki et al., 1989) versus $f_{up,man}$ = 0.114 (Taylor and Turner, 1981)], the $K_{p,i}$ values for the human WB-PBPK model were expressed as:

$$K_{p,i} = K_{p,SS,i} \cdot \frac{f_{up,man}}{f_{up,rat}} \cdot SF_{man} \tag{S10}$$

Based on Eqs. (S9) and (S10), the WB-PBPK model (i.e., Eqs. (S7) and (S8)) was fitted to the IV PK data in mice and humans (Table 1), which yielded the SF values of 1.49 (CV% = 15.7%) for mice

and 0.373 (13.5%) for humans. The tissue-to-plasma ‘concentration ratios’ calculated by Eqs. (S1), (S9) and (S10), which reflect drug depletion, were considered directly applicable in Eqs. (S7) and (S8), since the current WB-PBPK model assumed the central elimination kinetics.

1.2 Mathematical descriptions of the lumped PBPK models

Following the establishment of the WB-PBPK models, the WB-PBPK models for mice, rats, and humans were structurally reduced to the mPBPK model forms by lumping of the physiological tissues, resulting in the slowly equilibrating group (SEG) and rapidly equilibrating group (REG). The progressive tissue lumping was conducted until the model-derived terminal slope was kept less than 1% (0.530% for mice, 0.292% for rats, and 0.200% for humans).

The governing equations for the lumped model are given by Eqs. (S11) to (S13):

$$V_B \cdot R_B \cdot \frac{dC_p}{dt} = Input - Q_{SEG} \cdot R_B \cdot \left(C_p - \frac{C_{SEG}}{K_{p,SEG}}\right) - Q_{REG} \cdot R_B \cdot \left(C_p - \frac{C_{REG}}{K_{p,REG}}\right) - CL \cdot C_p \quad \text{(S11)}$$

$$V_{REG} \cdot \frac{dC_{REG}}{dt} = Q_{REG} \cdot R_B \cdot \left(C_p - \frac{C_{REG}}{K_{p,REG}}\right) \quad \text{(S12)}$$

$$V_{SEG} \cdot \frac{dC_{SEG}}{dt} = Q_{SEG} \cdot R_B \cdot \left(C_p - \frac{C_{SEG}}{K_{p,SEG}}\right) \quad \text{(S13)}$$

The blood flows to the SEG (Q_{SEG}) and the REG (Q_{REG}) are calculated as:

$$Q_{SEG} = \sum_{i \in SEG} Q_{T,i} \quad \text{(S14)}$$

$$Q_{REG} = \sum_{i \in REG} Q_{T,i} \quad \text{(S15)}$$

The anatomical volumes of the SEG (V_{SEG}) and the REG (V_{REG}) are calculated as:

$$V_{SEG} = \sum_{i \in SEG} V_{T,i} \quad \text{(S16)}$$

$$V_{REG} = \sum_{i \in REG} V_{T,i} \quad \text{(S17)}$$

The K_p values for the SEG ($K_{p,SEG}$) and the REG ($K_{p,REG}$) are calculated as:

$$K_{p,SEG} = \frac{\sum_{i \in SEG} V_{T,i} K_{p,i}}{\sum_{i \in SEG} V_{T,i}} \quad \text{(S18)}$$

$$K_{p,REG} = \frac{\sum_{i \in REG} V_{T,i} K_{p,i}}{\sum_{i \in REG} V_{T,i}} \quad \text{(S19)}$$

According to the principle of the progressive tissue lumping (Jeong et al., 2022a; b), the cumulative sums of the apparent distribution volumes ($\sum V_{T,i} K_{p,i}$) and the apparent distributional clearances ($\sum Q_{T,i} R_B$) were sequentially computed, starting from the tissue with the longest MTT (Eq. (15)). These bottom-up physiological metrics were then quantitatively compared with the top-down mPBPK parameter estimates for Tissue 1 ($V_1 K_p$ and $Q_{CO} f_{d1} R_B$) and Tissue 2 ($V_2 K_p$ and $Q_{CO} f_{d2} R_B$) derived from the individual fitting analysis.

2 Supplementary Figures and Tables

2.1 Supplementary Figures

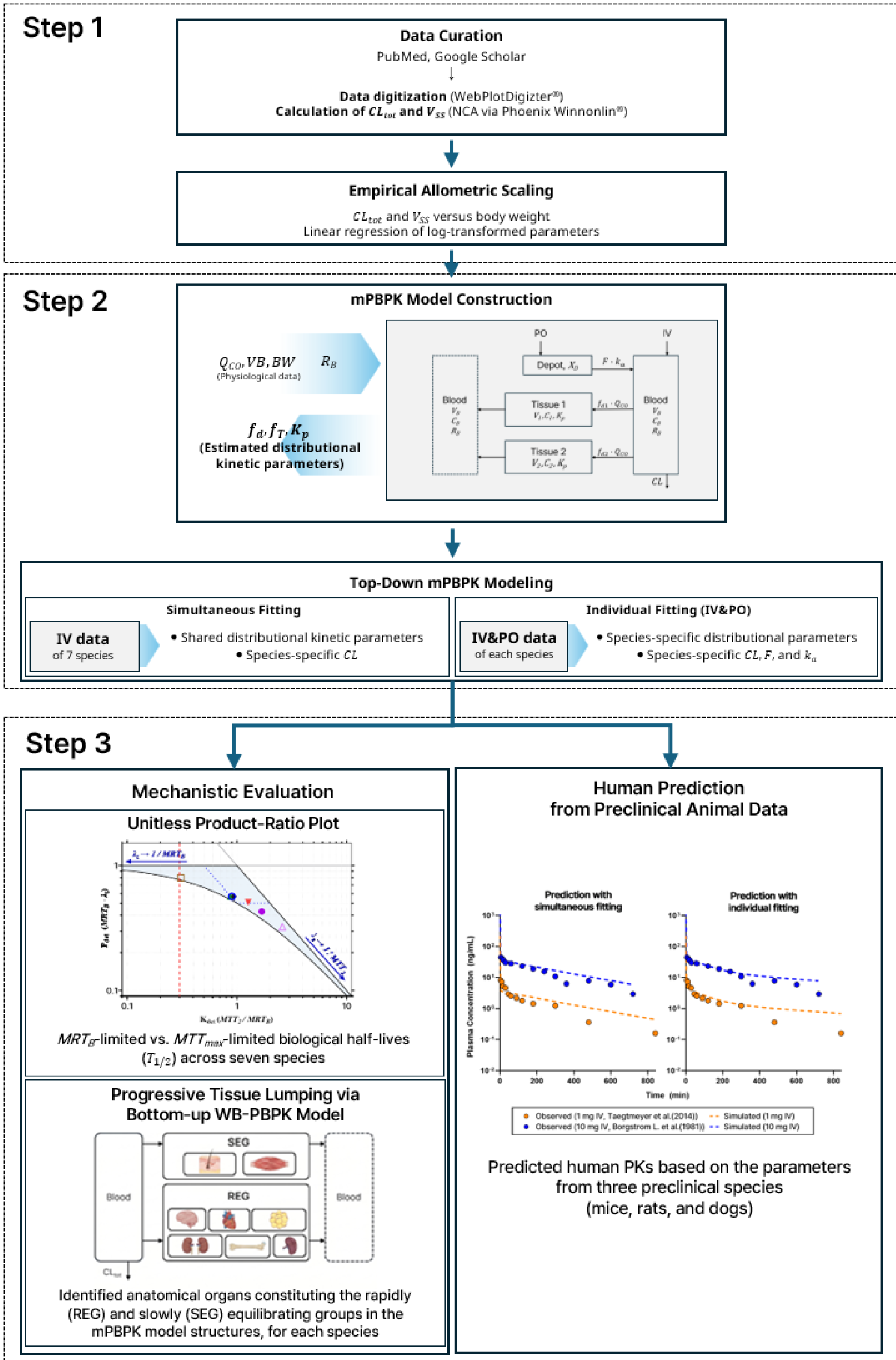

Supplementary Fig. S1. Overall workflow for the interspecies PK analysis of propranolol

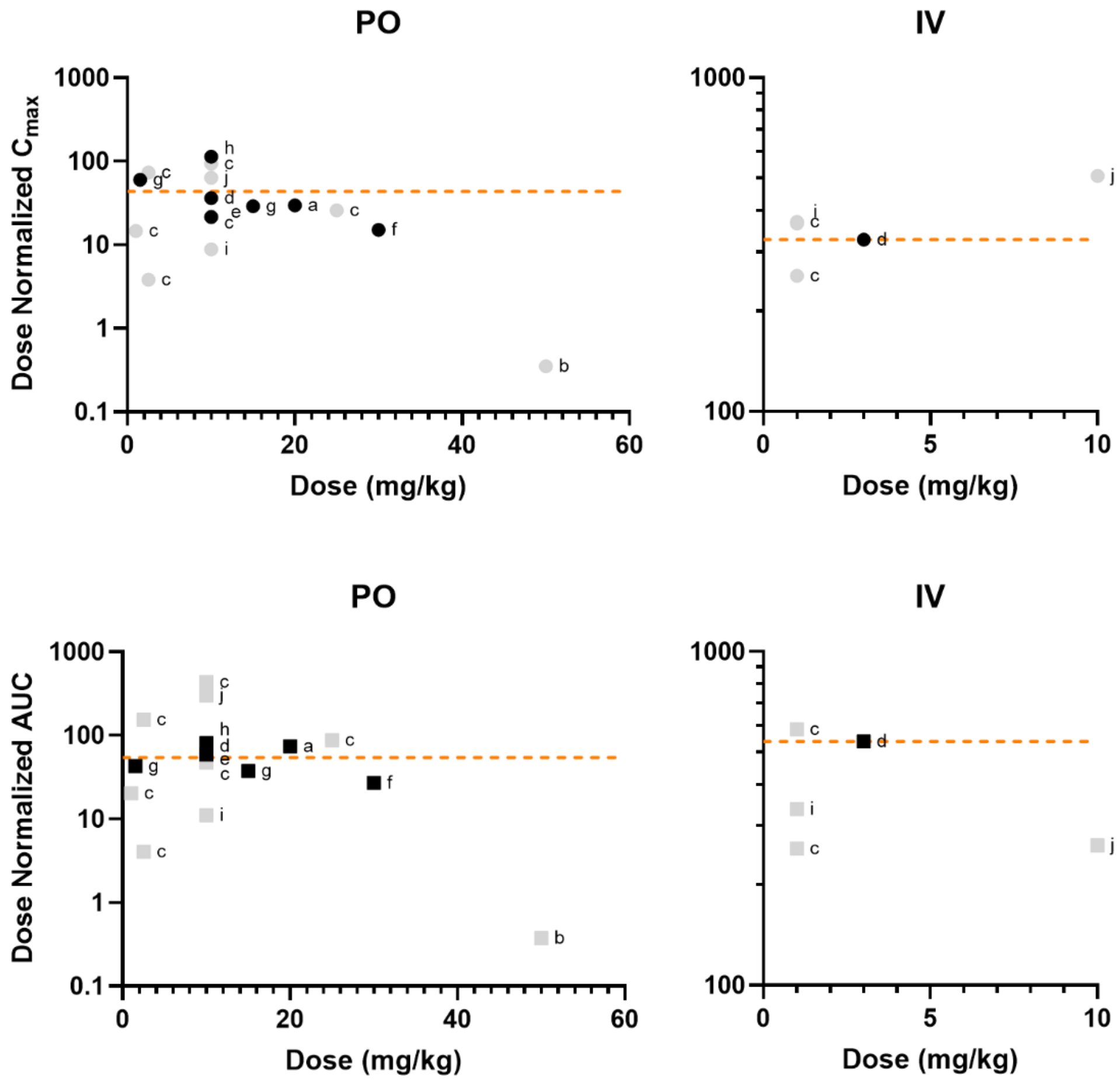

Supplementary Fig. S2. Summary of the literature-reported plasma PK data of propranolol after oral (PO; left panels) and IV (right panels) administration in rats, in terms of dose-normalized C_{max} (PO) and C_0 (IV) (upper panels), and dose-normalized AUC (lower panels) across doses (mg/kg). Closed symbols indicate datasets included in model fitting, whereas gray symbols indicate excluded datasets. Orange dashed lines are the mean values of datasets included in model fitting.

[a]Al Shaker et al. (2017); [b]Wenbin et al. (2015); [c]Komura et al. (2005); [d]Lee and Ku (1999); [e]Yoshigae et al. (1998); [f]Laethem et al. (1995); [g]Vercruysse et al. (1993); [h]Chow and Lalka (1993); [i]Vermeulen et al. (1992); [j]Shibasaki et al. (1989)

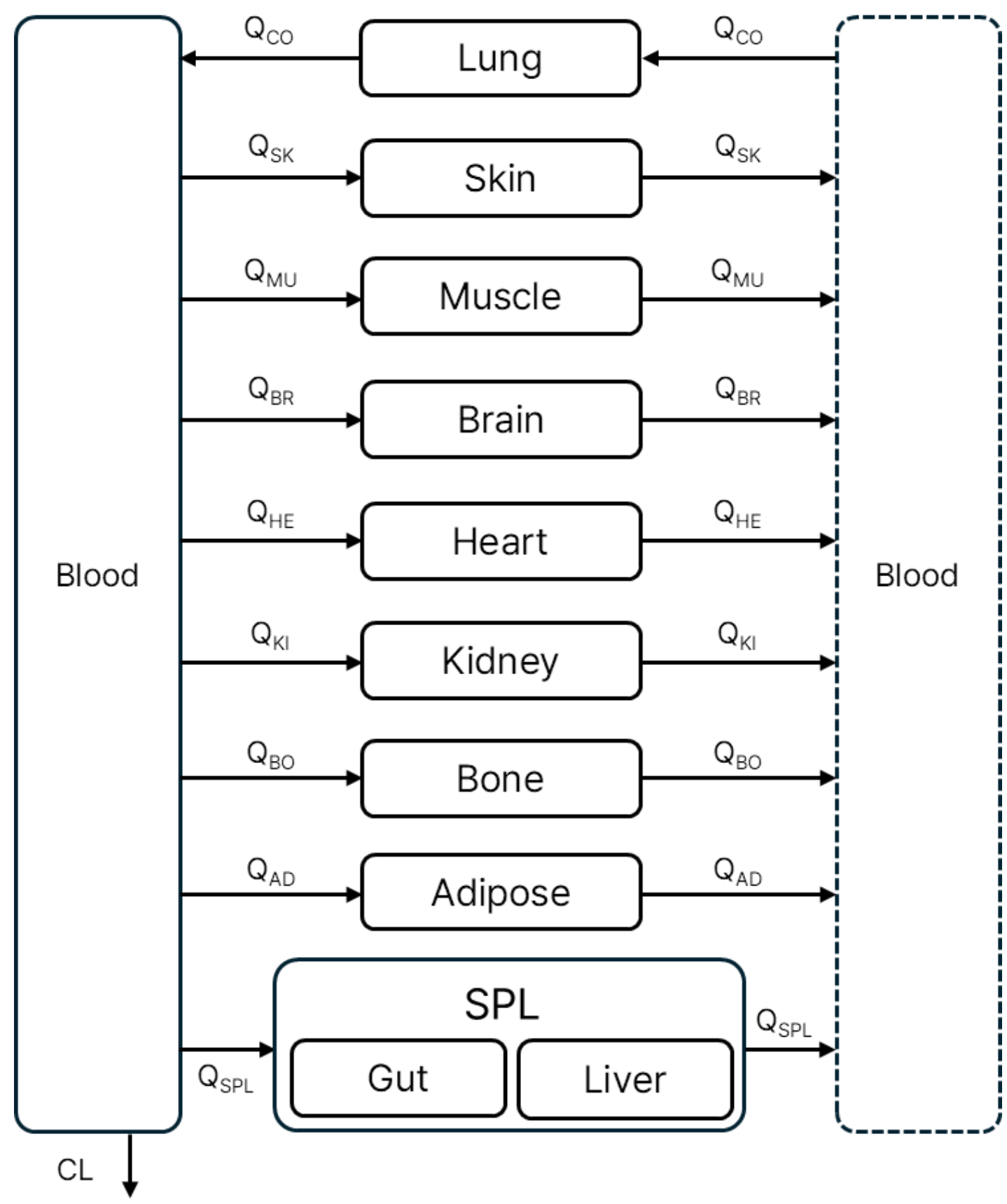

Supplementary Fig. S3. Schematic diagram of the WB-PBPK model developed for propranolol PKs in rats, mice, and humans. Detailed model structure, equations, and symbol definitions are provided in the Supplementary Material.

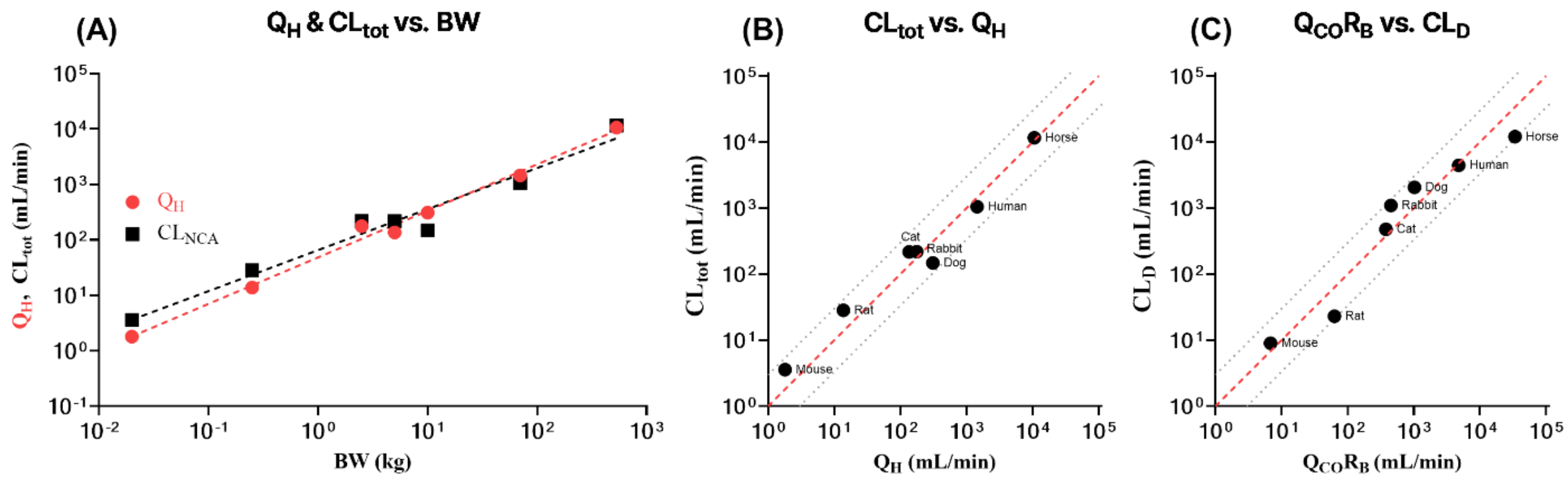

Supplementary Fig. S4. Cross-species comparison of propranolol clearance with physiological blood flow. (A) Hepatic blood flow (Q_H) and the reported total clearance (CL_{tot}; calculated from NCA if not reported) versus body weight. (B) CL_{tot} versus Q_H. (C) Total distributional clearance (CL_D), calculated from tri-exponential fitting (Supplementary Table S5), versus the apparent cardiac plasma flow ($Q_{CO}R_B$). Red dashed lines indicate identity, and gray dotted lines indicate three-fold limits.

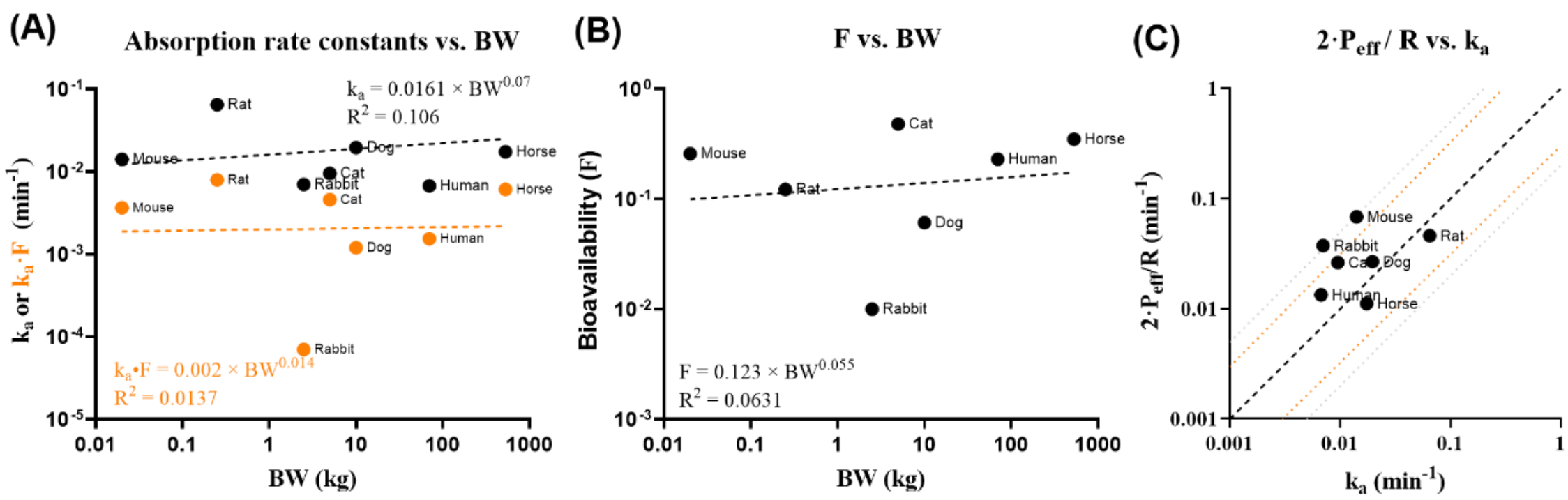

Supplementary Fig. S5. Relationships of oral absorption kinetic parameters of propranolol derived from individual fittings against body weight (BW) across seven species. (A) Oral absorption rate constants (k_a and $F \cdot k_a$) and (B) oral bioavailability (F) were plotted against BW. Dashed lines and equations represent the best-fit log-log linear regressions. (C) The estimated k_a values were compared with the values predicted using the equation (k_a=$2 \cdot P_{eff}/R$), where P_{eff} is the effective intestinal permeability and R is the gut radius (Jeong and Jusko, 2021). The reported value of $P_{eff,human}$ was 280 × 10^{-6} cm/s (Lennernas, 1997) and the same value was used for horses and dogs. Using $P_{eff,human}$, the $P_{eff,rat}$ value was calculated via the following empirical relationship ($P_{eff,human} = 3.6 \times P_{eff,rat} + 0.03 \times 10^{-4}\ cm/s$; (Fagerholm et al., 1996)). The same value was used for mice, rats, rabbits, and cats.

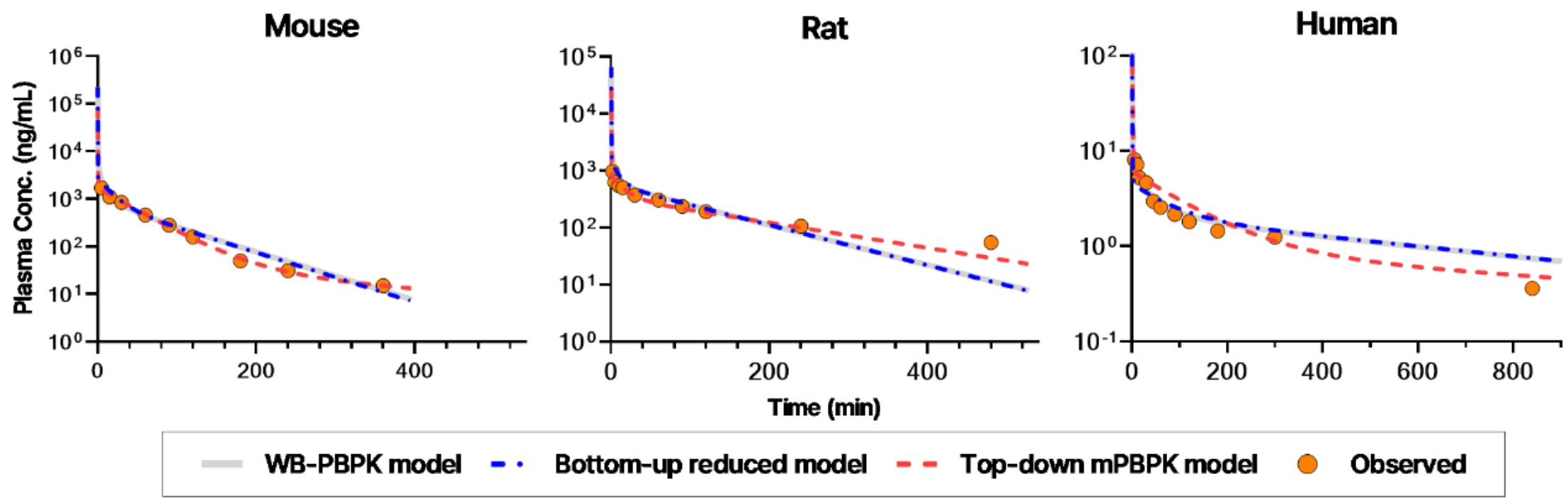

Supplementary Fig. S6. Comparisons among the simulated and observed plasma PK profiles after an IV bolus administration in mice (16 mg/kg), rats (3 mg/kg) and in humans (1 mg). The nine-tissue WB-PBPK model (gray solid lines), the reduced model (blue dashed lines), and top-down mPBPK model (red dashed lines) represent the model predictions, while the orange circles indicate the *in vivo* observed data in each species.

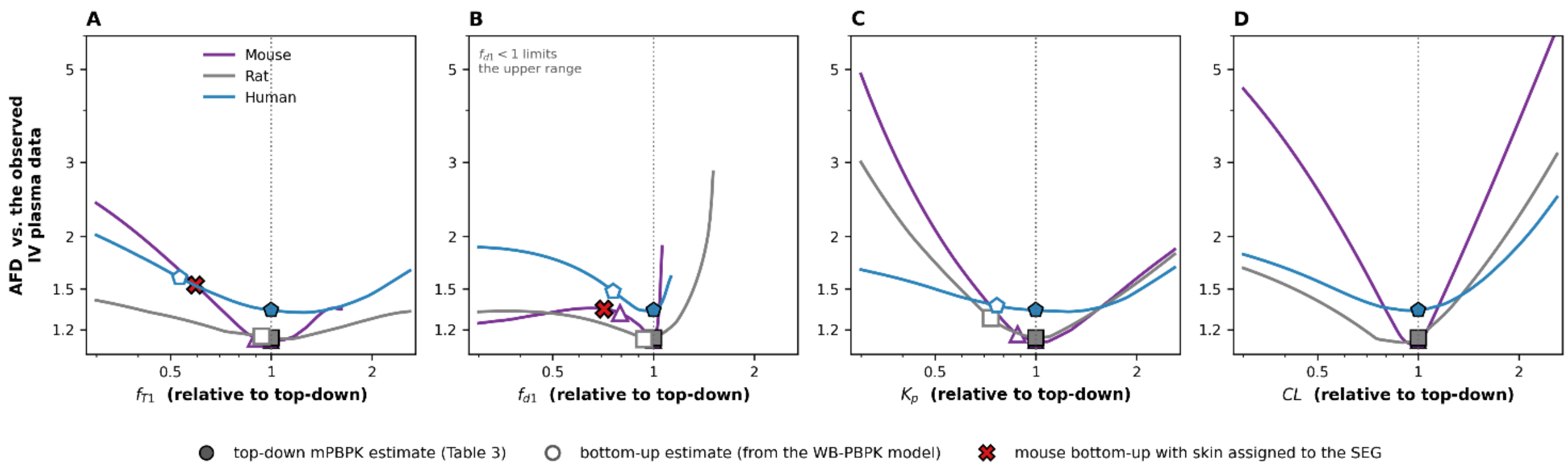

Supplementary Fig. S7. Local sensitivity of the fitted (top-down) mPBPK model parameters evaluated against the observed IV PK data. Model discrepancy was quantified as the average fold deviation (AFD) calculated as $AFD = 2^{\frac{1}{t_{last}}\int_0^{t_{last}}\left|log_2\left(\frac{C_{mPBPK}(t)}{C_{observed}(t)}\right)\right|dt}$, where C_{mPBPK} and $C_{observed}$ denote the model-predicted and observed plasma concentrations. (A-D) Model sensitivity to f_{T1}, f_{d1}, K_p, and CL, expressed relative to their top-down estimates (Table 3), with the remaining parameters fixed. Filled symbols indicate the top-down estimates, and open symbols indicate the bottom-up estimates obtained by progressive lumping of the WB-PBPK models. The red crosses in (A) and (B) indicate mouse bottom-up estimates with alternative assignment of the skin (a borderline tissue during tissue lumping, See discussion) to the SEG. The upper range of f_{d1} was constrained below 1.

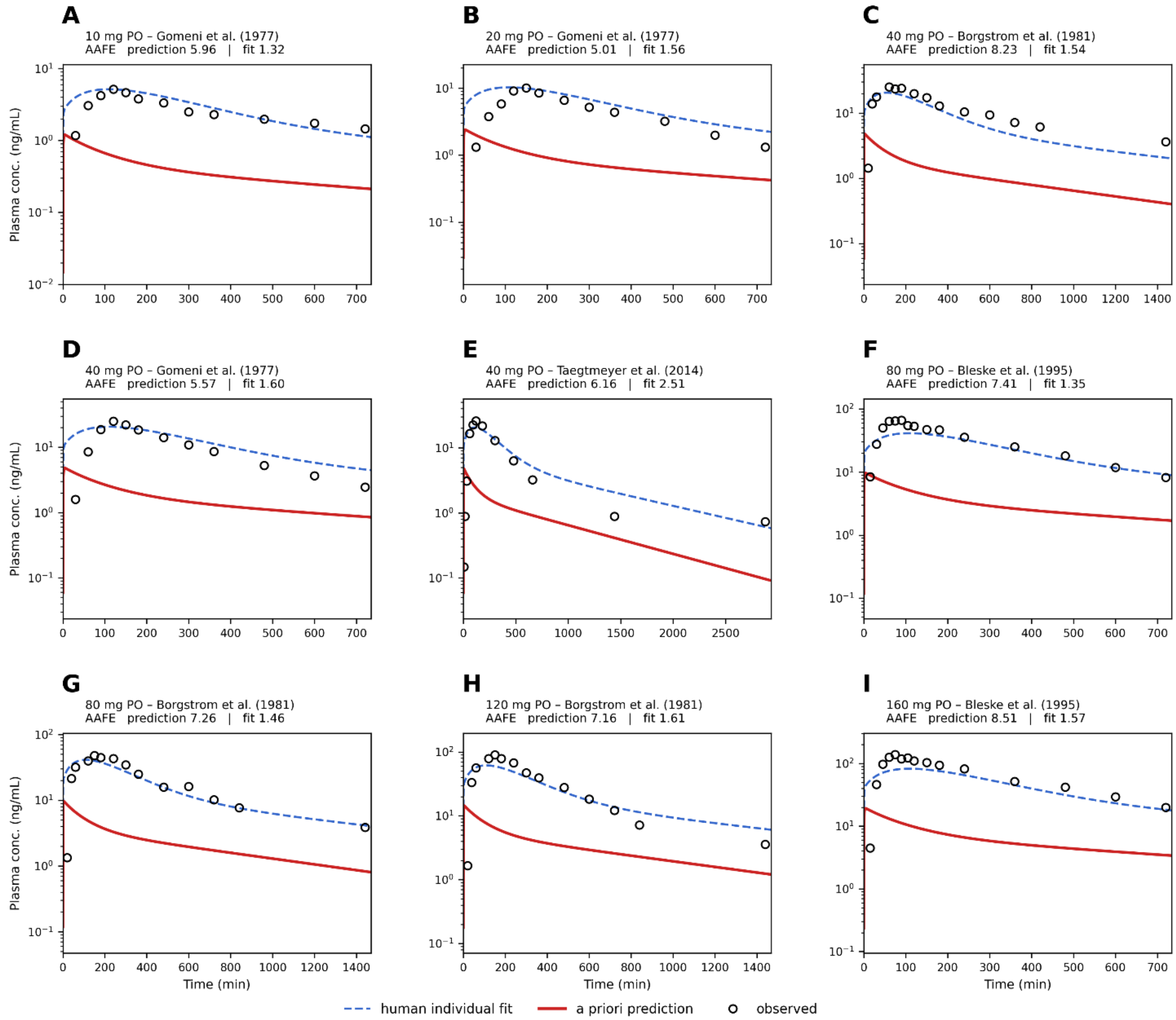

Supplementary Fig. S8. *A priori* prediction of human oral PKs of propranolol from three preclinical species. Observed and predicted plasma concentration–time profiles are shown for single oral doses of (A) 10 mg and (B) 20 mg (Gomeni et al., 1977); (C) 40 mg (Borgstrom et al., 1981); (D) 40 mg (Gomeni et al., 1977); (E) 40 mg (Taegtmeyer et al., 2014); (F) 80 mg (Bleske et al., 1995); (G) 80 mg and (H) 120 mg (Borgstrom et al., 1981); and (I) 160 mg (Bleske et al., 1995). Open circles indicate the observed data. Solid red lines indicate the a priori predictions obtained using k_a and F extrapolated from the log–log regressions against body weight in mice, rats, and dogs, together with the interspecies distribution parameters and the allometrically scaled CL. Dashed blue lines indicate the profiles obtained using the individually fitted human parameters (Table 3). The AAFE values for each prediction are given in the panel headings; the pooled AAFE across all nine datasets was 6.81 for the *a priori* prediction and 1.58 for the individual fit.

2.2 Supplementary tables

Supplementary Table S1. Summary of the *in vivo* PK data for propranolol across multiple species.

Species	Strain / Study Population	Sex	Body Weight (kg)	Dosing Route	Dose (mg/kg)	Dosed Isomer	Assay	Literature-Reported Values			NCA Values			Use in the present analysis	Reference
								CL (mL/min/kg)	CL/F (mL/min/kg)	V_{SS} (L/kg)	CL (mL/min/kg)	CL/F (mL/min/kg)	V_{SS} (L/kg)		
Mouse	Swiss- Webster	M, F	0.02-0.025	IV	16	Racemate, R, S	Radio-immunoassay				179		12.0	S, I	(Levy et al., 1976)
Mouse	CD/1	-	-	PO	30	Racemate	Supercritical Fluid Chromatography -Tandem Mass Spectrometry					311		I	(Chen et al., 2006)
Rat	Sprague-Dawley	M, F	0.220	PO	20	Racemate	RP-HPLC-UV		23.88 L/hr			389		S	(Al Shaker et al., 2017)
Rat	Wistar (in plain area)	M	0.200	PO	50	Racemate	LC/MS./MS		113.528 L/hr			4.45×10^{4}		Not used; outlying dose-normalised exposure	(Wenbin et al., 2015)
	Wistar (in high altitude)								21.077 L/hr			8.85×10^{3}			
Rat	Wistar		0.156	IV	1	Racemate	LC/MS	70.3		3.56	64.9		3.30	Not used; outlying dose-normalised exposure	(Komura et al., 2005)
	DA		0.330	IV				30.4		2.46	28.5		2.15		
	Wistar		0.156	PO	2.5							4.10×10^{3}			
					10							353			
					25							191			
	DA		0.330	PO	1							818			
					2.5							108			
					10							38.5			
Rat	Sprague-Dawley	M		IV	3	Racemate	HPLC-fluorescence	30.7		8.37	31.0		7.19	S, I	(Lee and Ku, 1999)

				PO	10				290.35			290		I	
	Sprague-Dawley with AKI			IV	3			29.7		8.80				S, I	
				PO	10				132.3					I	
Rat	Wistar	M	0.230-0.270	PO	2.5	Racemate	HPLC		547					I	(Yoshigae et al., 1998)
				PO	10				229			284		I	
Rat	Wistar	M	0.443	PO	30	Racemate	HPLC-fluorescence/DATAAN		610^{R}, $6.29\times10^{3\,S}$					I	(Laethem et al., 1994)
	Wistar, Endotoxin treated (increased AAG)		0.412	PO	30	Racemate			90^{R}, $1.95\times10^{3\,S}$					I	
Rat	Wistar		0.300	IV	1.5	Racemate	HPLC	26.9						S, I	(Vercruysse et al., 1993)
				PO	1.5				510.2			390		I	
					15				475.3			445		I	
Rat	Sprague-Dawley		0.260-0.375	PO	10	R	HPLC		198.4			206		Not used; outlying dose-normalised exposure	(Chow and Lalka, 1993)
	Sprague-Dawley glucose pretreatment			PO	10				273.2					Not used; non-healthy or non-standard condition	
Rat	Wistar (SPF) 3m/o		0.344	IV	1	Racemate	HPLC-fluorescence	76.7^{R}, 57.5^{S}			36.0^{R}, 68.9^{S}		1.96^{R}, 9.84^{S}	Not used; outlying dose-normalised exposure	(Vermeulen et al., 1992)
	Wistar (SPF) 12m/o		0.529					63.3^{R}, 48.7^{S}			30.6^{R}, 48.3^{S}		1.54^{R}, 8.57^{S}	Not used; non-healthy or non-standard condition	
	Wistar (SPF) 24m/o		0.592					55.1^{R}, 40.2^{S}			28.2^{R}, 59.6^{S}		2.08^{R}, 8.83^{S}		
	Wistar (SPF) 3m/o		0.344	PO	10				$1.68\times10^{3\,R}$, $9.05\times10^{3\,S}$			$8.73\times10^{4\,R}$, $4.21\times10^{5\,S}$		Not used; outlying dose-normalised exposure	
	Wistar (SPF) 12m/o		0.529						$1\times10^{3\,R}$, $6.22\times10^{3\,S}$			$5.15\times10^{4\,R}$, $3.52\times10^{5\,S}$		Not used; non-healthy or non-standard condition	
	Wistar (SPF) 24m/o		0.592						$1.11\times10^{3\,R}$, $5.63\times10^{3\,S}$			$4.03\times10^{4\,R}$, $2.07\times10^{5\,S}$			

Rat	Sprague-Dawley		0.250-0.300	IV	10	Racemate	Spectro fluorometry	76.2		5.98	63.6		7.68	Not used; outlying dose-normalised exposure	(Shibasaki et al., 1989)
				PO	10				335.7			55.3			
	Sprague-Dawley, cimetidine pretreatment			IV	10			47.1							
				PO	10				205.3						
Rat	Sprague-Dawley	M	0.250-300	IV	1.5	S	HPLC	53.5		6.51	51.4		4.25	Not used; outlying dose-normalised exposure	(Terao and Shen, 1984)
				PO	6				1875						
	Sprague-Dawley rats (Uranyl nitrate induced AKI)			IV	1.5			56.6		6.42				Not used; non-healthy or non-standard condition	
				PO	6				521.7						
Rabbit	New-Zealand	M	3±0.1	PO	50	Racemate	HPLC		$21.97\times10^{3\,R}$, $15.04\times10^{3\,S}$			$1.20\times10^{4\,R}$, $9.74\times10^{3\,S}$		I	(Laethem et al., 1995)
Rabbit	New-Zealand White	M	3-5	IV	1		HPLC	85.53		8.67	87.30		8.68	S, I	(al-Meshal et al., 1993)
Rabbit	New-Zealand White (Normal and HTN Mixed)	M	2.7	IV	0.55			3.13 L/hr		7.82 L	165		24.9	Not used; non-healthy or non-standard condition	(Corbo et al., 1989)
Cat	-	M	3-5	IV	0.5-1		HPLC-fluorescence	43.4		3.37	43.4		2.30	S	(Arendt et al., 1984)
Cat	-	F	-	IV	0.5		HPLC	28		1.72	23.5		1.60	S, I	(Jacobs et al., 1997)
				PO	5				101.75			46.1		I	
Dog	Mongrel	M, F	10-20	IV infusion	0.1 mg/kg/min	Racemate	GC/MS				14.8		3.09	S, I	(Walle et al., 1989)
Dog	Mongrel	M	25-30	PO	5	Racemate	HPLC		440^{R} 690^{S}			231^{R}, 315^{S}		I	(Laethem et al., 1995)
Dog	Mongrel	-	-	PO	3	Racemate	Stereoselective Reversed-Phase HPLC		120^{R} 205^{S}			120^{R}, 215^{S}		Not used; non-healthy or non-standard condition	(Schaefer et al., 1990)
	Mongrel (Partial gastrectomy)								95.5^{R} 180.7^{S}						
Human	Healthy	M, F	-	IV	1 mg		HPLC	1187 mL/min		4.4			244 L	S, I	(Taegtmeyer et al., 2014)

				PO	40 mg					3.57×10^3 mL/min	I	
Human	Healthy	M, F	65.5	PO	10 mg	Racemate	-		4.42×10^3 mL/min	4.75×10^3 mL/min	Not used; non-standard formulation or not PO/IV doses	(Wang et al., 2013)
				SL	10 mg	Racemate		1.87×10^3 mL/min				
Human	Coronary Heart Disease (preoperative)	M, F	75.4	PO	10 mg	Racemate	HPLC-fluorescence		9.2		Not used; non-healthy or non-standard condition	(Carmona et al., 2005)
	Patients after CABG with hypothermic cardiopulmonary bypass								10.7			
Human	Healthy	M, F	73.1	PO	80 mg	Racemate	HPLC-Spectrofluorometry		16.7	2.07×10^3 mL/min	Not used; non-healthy or non-standard condition	(Wojcicki et al., 2003)
	Obese normolipaemic		94.6						11.7			
	Obese hyperlipidaemic		105.7						10.0			
Human	Healthy	M, F	71	PO	80 mg	Racemate	HPLC-spectrofluorometry		40.3	2.31×10^3 mL/min	Not used; non-healthy or non-standard condition	(Wojcicki et al., 1999)
	Hypercholesterolemia		67.38						48.5			
	Hypertriglyceridemia		82.60						21.7			
	Mixed form of hyperlipidemia		88.27						48.3			
Human	Healthy Chinese	M	52	PO	80 mg	Racemate	HPLC-fluorescence		38.3	1.53×10^3 mL/min	Not used; non-healthy or non-standard condition	(Xie and Chen, 1995)
		F							18.3	944 mL/min		
Human	Healthy		25% within IBW	PO	80 mg IR	Racemate	HPLC-fluorescence		436 L/hr [R], 270 L/hr [S]	4.31×10^3 mL/min [R], 3.01×10^3 mL/min [S]	I	(Bleske et al., 1995)
				PO	160 mg IR				360 L/hr [R], 234 L/hr [S]	3.82×10^3 mL/min [R], 2.79×10^3 mL/min [S]	I	
				PO	160 mg SR						Not used; non-standard formulation or not PO/IV doses	

				IV	10 mg			76 L/hr ^{R}, 63 L/hr ^{S}		1.29×10^3 mL/min ^{R}, 1.01×10^3 mL/min ^{S}		304 L ^{R} 290 L ^{S}	I	
Human	Healthy	M, F	57-80	PO	40 mg	Racemate	GC-ECD		4.33×10^3 mL/min		3.24×10^3 mL/min		Not used; non-healthy or non-standard condition	(Vanakoski and Seppala, 1995)
	Healthy, After Sauna								3.90×10^3 mL/min					
Human	Healthy	M, F		PO	40 mg	Racemate	HPLC		6623 mL/min		4.63×10^3 mL/min		Not used; non-healthy or non-standard condition	(Greenblatt et al., 1993)
Human	Healthy (younger)	M, F	61.0	PO (9 am)	20 mg	Racemate	HPLC		56.2				Not used; non-healthy or non-standard condition	(Shiga et al., 1993)
				PO (9 pm)					66.9					
	Healthy (older)	M, F	53.0	PO (9 am)					46.6					
				PO (9 pm)					52.5					
Human	Healthy Black		79.7	PO	80 mg	Racemate			27.6 ^{R} 42.3 ^{S}				Not used; non-healthy or non-standard condition	(Johnson and Burlew, 1992)
	Healthy White		81.3						27.6 ^{R} 27.7 ^{S}					
Human	Healthy		45-66	PO	40 mg	Racemate	HPLC				1.55×10^3 mL/min		Not used; IV and PO data were not presented together in the study.	(Bano et al., 1991)
Human	Healthy		63.2	PO	10 mg	Racemate	HPLC				7.53×10^3 mL/min		Not used; IV and PO data were not presented together in the study.	(Yasuhara et al., 1990)
			60.8		20 mg						8.06×10^3 mL/min			
Human	Healthy		77	PO	80 mg	Racemate	HPLC				4.34×10^3 mL/min		Not used; IV and PO data were not presented together in the study.	(Schoors et al., 1990)
Human	Healthy		76.7	PO	80 mg	Racemate			34.0 ^{R} 23.9 ^{S}		1.84×10^3 mL/min ^{R} 1.22×10^3 mL/min ^{S}		Not used; IV and PO data were not presented together in the study.	(Hunt et al., 1990)
Human	Healthy		55-83	PO	20 mg	Racemate					7.26×10^3 mL/min		Not used; IV and PO data were not presented together in the study.	(Tateishi et al., 1989)

Human	Healthy			PO	80 mg							1.36×10^{3} mL/min		Not used; IV and PO data were not presented together in the study.	(McCourty et al., 1988)
Human	Healthy		75	PO	160 mg	Racemate	HPLC-fluorescence		503			2.71×10^{4} mL/min		Not used; IV and PO data were not presented together in the study.	(Straka et al., 1987)
Human	Healthy			PO	80 mg	Racemate			2.13 L/min			1.44×10^{3} mL/min		Not used; IV and PO data were not presented together in the study.	(Henry et al., 1987)
Human	Healthy			PO	80 mg	Racemate	HPLC-fluorescence					470 mL/min		Not used; IV and PO data were not presented together in the study.	(Williams et al., 1986)
Human	Healthy	M, F	66.8	IV infusion for 10 min	10 mg	Racemate	HPLC-fluorescence	11.9		3.0	651 mL/min			Not used; non-healthy or non-standard condition	(Bowman et al., 1986)
				PO	40 mg				3.0 L/min						
	Obese	M, F	136.5	IV infusion for 10 min	10mg			6.1		2.6					
				PO	40 mg				2.4 L/min						
Human	EM	M	-	PO	80 mg	Racemate	HPLC	3.21 L/min						Not used; non-healthy or non-standard condition	(Lennard et al., 1984)
	PM							3.21 L/min							
Human	Healthy	M, F	62-92	IV infusion 1mg/min	0.1	Pseudoracemate	GC/MS	1.21 L/min ^{R} 1.03 L/min ^{S}		4.82 ^{R} 4.08 ^{S}	12.0 ^{R} 10.2 ^{S}		3.01 ^{R} 2.65 ^{S}	I	(Olanoff et al., 1984)
Human	Healthy pregnant	F	66.9	IV infusion for 10 min	10 mg	Racemate	GLC-ECD	11.3		1.9				Not used; non-healthy or non-standard condition	(O'Hare et al., 1984)
				PO	120 mg				23.2						
	Healthy (Postnatal)		58.7	IV infusion for 10 min	10 mg			10.6		2.7					
				PO	120 mg				17.8						
Human	Healthy	M	-	PO	80 mg	Racemate	HPLC-fluorescence		3.12 L/min			2.89×10^{4} mL/min		Not used; IV and PO data were not	(Gangji et al., 1984)

														presented together in the study.	
Human	Moderate HTN	M, F	-	PO	160 mg qd	Racemate	-		1.89 L/min<br>1.44 L.min					Not used; non-healthy or non-standard condition	(Serlin et al., 1983)
					80 mg bid				2.25 L/min<br>1.28 L/min						
					160 mg qd	Racemate, Long Acting			4.76 L/min<br>2.86 L/min						
Human	Pregnant in 1st trimester with HTN	F	60.6	PO	0.66	Racemate	-	-						Not used; non-healthy or non-standard condition	(Smith et al., 1983)
	Pregnant in 2nd trimester with HTN		54/4		0.544										
	Pregnant in 3rd trimester with HTN		67.4		0.647										
	Pregnant >3 mths p-p with HTN		65.0		0.650										
Human	Healthy	M	-	PO	40mg q6h	Racemate	HPLC-fluorescence		3.97 L/min					Not used; non-healthy or non-standard condition	(Charles et al., 1982)
					160 mg qd	Racemate, Long Acting			3.89 L/min						
Human	Healthy young non-smokers	M	76	PO	80 mg tid	Racem41 0ate	HPLC-fluorescence		53.6					Not used; non-healthy or non-standard condition	(Hitzenberger et al., 1982)
	Healthy young smokers		74						46.4						
	Healthy old non-smokers		79						17.3						
	Healthy old smokers		73						21.9						
Human	Thyrotoxic patients	M, F	53-78 (Mean 66)	PO	160 mg	Racemate	Fluorometry		5.66 L/min					Not used; non-healthy or non-standard condition	(Aro et al., 1982)
	Thyrotoxic patients after achieving euthyroidism								3.01 L/min						
Human	Healthy		79.4	IV infusion for 30min	10 mg	Racemate	GLC/EC	917 mL/min		6	58.4 L/hr		280 L	I	(Borgstrom et al., 1981)
				PO	40 mg				2103.6 mL/min					I	
					80 mg				3114.0 mL/min					I	

					120 mg				3176.2 mL/min		I	
Human	Hyperthyroid	M, F	39-61 (Mean 49.3)	PO	40 mg	Racemate	GLC		37.7<br>24.0 ^{S}	14.8<br>9.4 ^{S}	Not used; non-healthy or non-standard condition	(Tawara et al., 1981)
	Hyperthyroid patients after antithyroid drug Tx								32.5<br>20.3 ^{S}	11.5<br>7.2 ^{S}		
Human	Uncomplicated essential HTN	M, F		PO	80 mg	Racemate	Fluorimetry		2.65 L/min		Not used; non-healthy or non-standard condition	(Krediet et al., 1980)
Human	Healthy	M, F	-	PO	160 mg	Racemate	Spectrofluorimetry		1.49 L/min		Not used; non-healthy or non-standard condition	(Leahey et al., 1980)
					160 mg Long Acting				2.17 L/min			
					160 mg for 8 days				1.47 L/min			
					160 mg Long Acting for 8 days				2.15 L/min			
Human	Healthy	M	64.5	IV infusion for 20min	0.1 mg/kg	Racemate	GC	91.7		4.8	I	(Gomeni et al., 1977)
				PO	0.155 (10 mg)				51.6		I	
					0.310 (20 mg)				92.3		I	
					0.620 (40 mg)				85.4		I	
Human	Healthy	M	71	PO	40 mg	Racemate	GC		47.7		Not used; non-healthy or non-standard condition	(Bianchetti et al., 1976)
	CKD	M, F	60						9.95			
	Patients undergoing regular dialysis Tx (After dialysis day)	M, F	57						36.6			
	(On dialysis day)								36.2			
Human	Permanent HTN	M, F	68.4	IV	0.2	Racemate	Spectrofluorimetry	12.3		3.01	Not used; non-healthy or non-standard condition	(Weiss et al., 1976)
	Borderline HTN	M, F	72.3					19.4		2.87		

Human	Healthy			PO	80 mg									Not used; non-healthy or non-standard condition	(Lowenthal et al., 1974)
	CKD														
Horse	Gelding	M	490-600	IV	0.2	GC/MS	1.37		3.05	1.31			2.79	S, I	(Aramaki et al., 2000)
				PO	1			813						I	

S Dataset used for the simultaneous fitting of the IV data across the seven species (Table 1). I Dataset used for the individual fitting of the IV and oral data of the corresponding species (Table 1). For rodents, datasets whose dose-normalised exposure deviated substantially from the remainder were excluded (Supplementary Fig. S2). For humans, all IV datasets obtained in healthy subjects were retained, whereas oral datasets were retained only when IV data had been obtained in the same study, so that F could be estimated within a single study population. Datasets obtained under non-standard physiological conditions (high altitude, endotoxin treatment, renal impairment, thyroid disease, obesity, pregnancy, co-medication) or with non-standard formulations (sustained release, sublingual) or dosing regimens (multiple dosing) are tabulated for completeness only and were not used for model development.

Supplementary Table S2. Physiological parameters used for mPBPK modeling of propranolol PK in seven species.

Species	Body Weight (BW, kg)	Blood Volume (V_B, mL)	Cardiac Output (Q_{CO}, mL/min)	Hepatic Blood Flow (Q_H, mL/min)
Mouse	0.02	1.7	8	1.8
Rat	0.25	13.5	74	13.8
Rabbit	2.5	165	530	177
Cat	5	$BW \times 55$ [a]	$BW \times 89$ [b]	$BW \times 27.1$ [c]
Dog	10	900	1200	309
Human	70	5200	5600	1450
Horse	530	26000	39800	$BW \times 20$ [d]

Values obtained from Davies and Morris (1993) unless otherwise specified: [a]Aravindh and Jacob (2021); [b]Bartorelli and Gerola (1963); [c]Lautt (1977); [d]Dyke et al. (1998)

Supplementary Table S3. Summary of the physiological information (V_T and Q_T) used for construction of the WB-PBPK model for propranolol in mice, rats, and humans

	Mouse		Rat		Human	
**Tissue**	Q_T (mL/min)	V_T (mL)	Q_T (mL/min)	V_T (mL)	Q_T (mL/min)	V_T (mL)
Skin	0.464	3.31	4.29	47.6	336	2,600
Muscle	1.27	7.68	20.6	101	1,220	28,000
Brain	0.264	0.330	1.48	1.43	638	1,400
SPL[b]	1.29	1.94	13.5	15.9	1,270	3,000
Gut	0.336	0.844	7.50	6.75	1,100	1,200
Liver	1.29	1.10	13.5	9.15	1,270	1,800
Bone	0.880	2.15	9.03	18.2	235	10,000
Kidney	0.728	0.334	10.4	1.83	980	308
Heart	0.528	0.100	3.77	0.825	224	329
Adipose	0.560[a]	1.90	5.18	6.64	291	15,000

Parameters were obtained from Brown et al. (1997) unless otherwise specified. [a]The fraction of the cardiac output to the tissue was assumed identical between rats and mice. [b] Eqs. (S4) and (S5)

Supplementary Table S4. Summary of the distribution kinetic parameters calculated for the WB-PBPK model in rats, mice, and humans. Tissues for which the tissue-to-plasma partition coefficient data for each enantiomer ($K_{pu,R}$ and $K_{pu,S}$) were not available (e.g., adrenal glands, pancreas, and spleen) were excluded from model construction.

Tissues	$K_{pu,R}$ [a]	$K_{pu,S}$ [a]	ER	$\frac{C_{T,SS,R}}{C_{p,SS,R}}$ [b]	$\frac{C_{T,SS,S}}{C_{p,SS,S}}$ [b]	$K_{p,SS}$ [c]	K_p (mouse) [d]	K_p (human) [d]
Adipose	38	19	-	0.65	2.41	1.27	1.89	1.69
Bone	82	53	-	1.39	6.73	3.27	4.88	4.36
Brain	383	281	-	6.51	35.7	16.8	25.0	22.3
Gut	369	182	-	6.27	23.1	12.2	18.2	16.2
Heart	227	124	-	3.86	15.7	8.05	12.0	10.7
Kidney	364	278	-	6.19	35.3	16.5	24.5	21.9
Liver	327	231	0.73 [e]	20.6	109	3.76	5.61	5.01
Lungs	1426	1037	-	24.2	132	62.1	92.5	82.6
Muscles	111	74	-	1.89	9.40	4.53	6.76	6.03
Skin	64	41	-	1.09	5.21	2.54	3.78	3.38
Testes	212	150	-	3.60	19.1	9.05	13.5	12.0
Thyroid	290	150	-	4.93	19.1	9.91	14.8	13.2

[a] Rodgers et al. (2005)

[b] Eqs. (S2) and (S3)

[c] Eq. (S1), applied for rats

[d] Eq. (S9) for mice and Eq. (S10) for humans

[e] Singh et al. (1991)

Supplementary Table S5. Summary of the parameters obtained from the tri-exponential curve fitting, and comparison between the total distributional clearances (CL_D) of propranolol and the cardiac output ($Q_{CO}R_B$; with respect to the plasma flow) across species.

Species (BW, kg)	Source	C_0 (ng/mL)	C_1 (ng/mL)	λ_1 (min^{-1})	C_2 (ng/mL)	λ_2 (min^{-1})	C_3 (ng/mL)	λ_3 (min^{-1})	CL_D (mL/min)	$Q_{CO} \cdot R_B$ (mL/min)
Mouse (0.02)	(Levy et al., 1976)	221,000	220,000	8.20	1,630	0.0217	37.3	0.00263	9.00	6.80
Rat (0.25)	(Lee and Ku, 1999)	65,400	64,700	2.59	412	0.0206	211	0.00282	23.1	62.9
Rabbit (2.5)	(al-Meshal et al., 1993)	17,800	17,600	9.32	179	0.0723	73.5	0.00817	1,100	451
Cat (5)	(Arendt et al., 1984)	12,800	12,400	2.97	312	0.180	139	0.0146	477	378
Dog (10)	(Walle et al., 1989)	58,800	55,300	3.08	2,460	0.0529	1,020	0.00492	2,060	1,020
Horse (530)	(Aramaki et al., 2000)	4,800	4,710	0.920	59.1	0.0160	23.5	0.00501	12,100	33,800
Human (70)	(Taegtmeyer et al., 2014)	226	217	1.22	6.59	0.0399	2.26	0.00218	4,460	4,760

Plasma PK data were fitted to a tri-exponential function ($C_p(t) = C_1 e^{-\lambda_1 t} + C_2 e^{-\lambda_2 t} + C_3 e^{-\lambda_3 t}$). The initial plasma concentration (C_0) was fixed as $Dose/(V_B \cdot R_B)$, which renders C_3 estimated as a secondary parameter ($C_3 = C_0 - C_1 - C_2$). For dogs, the slopes and intercepts were determined considering the zero-order infusion kinetics (duration =45 min).

Total distributional clearance (CL_D) was calculated as: $CL_D = Dose \cdot \left(\frac{\sum C_i \lambda_i}{C_0^2} - \frac{1}{AUC}\right)$ where $AUC = \sum C_i/\lambda_i$ (Veng-Pedersen, 1984; Gillespie and Veng-Pedersen, 1985)

3 Supplementary Data

3.1 ADAPT5 code used for simultaneous fitting

```
***********************************************************************
C                           ADAPT                                     *
C                         Version 5                                   *
C**********************************************************************
C                                                                     *
C                           MODEL                                     *
C                                                                     *
C    This file contains Fortran subroutines into which the user      *
C    must enter the relevant model equations and constants.          *
C    Consult the User's Guide for details concerning the format for  *
C    entered equations and definition of symbols.                    *
C                                                                     *
C       1. Symbol-  Parameter symbols and model constants             *
C       2. DiffEq-  System differential equations                     *
C       3. Output-  System output equations                           *
C       4. Varmod-  Error variance model equations                    *
C       5. Covmod-  Covariate model equations (ITS,MLEM)              *
C       6. Popinit- Population parameter initial values (ITS,MLEM)    *
C       7. Prior -  Parameter mean and covariance values (ID,NPD,STS) *
C       8. Sparam-  Secondary parameters                              *
C       9. Amat  -  System state matrix                               *
C                                                                     *
C**********************************************************************

C######################################################################C

        Subroutine SYMBOL
        Implicit None

        Include 'globals.inc'
        Include 'model.inc'

CC
C----------------------------------------------------------------------C
C   Enter as Indicated                                                 C
C----c-----------------------------------------------------------------C

      NDEqs   =  21   ! Enter # of Diff. Eqs.
      NSParam =  10   ! Enter # of System Parameters.
      NVparam =  2   ! Enter # of Variance Parameters.
      NSecPar =  4   ! Enter # of Secondary Parameters.
```

```
      NSecOut =  0  ! Enter # of Secondary Outputs (not used).
      Ieqsol  =  1  ! Model type: 1 - DIFFEQ, 2 - AMAT, 3 - OUTPUT only.
      Descr   = ' Universial Fitting for 7 Species  '

CC
C----------------------------------------------------------------------C
C   Enter Symbol for Each System Parameter (eg. Psym(1)='Kel')         C
C----c-----------------------------------------------------------------C

      PSym(1) = 'fd1temp'
      Psym(2) = 'FVTtemp'
      Psym(3) = 'Kpsum'
      Psym(4) = 'CLtotM'
      Psym(5) = 'CLtotR'
      Psym(6) = 'CLtotRB'
      Psym(7) = 'CLtotC'
      Psym(8) = 'CLtotD'
      Psym(9) = 'CLtotHO'
      Psym(10) = 'CLtotH'

CC
C----------------------------------------------------------------------C
C   Enter Symbol for Each Variance Parameter {eg: PVsym(1)='Sigma'}    C
C----c-----------------------------------------------------------------C

      PVsym(1)='Sigma'
      PVsym(2)='Intercept'

CC
C----------------------------------------------------------------------C
C   Enter Symbol for Each Secondary Parameter {eg: PSsym(1)='CLt'}     C
C----c-----------------------------------------------------------------C

      PSsym(1)='fd1sym'
      PSsym(2)='FVTsym'
      PSsym(3)='fd2sym'
      PSsym(4)='FVT2sym'


C----------------------------------------------------------------------C
C----------------------------------------------------------------------C
C
        Return
        End
```

```
C#######################################################################C

        Subroutine DIFFEQ(T,X,XP)
        Implicit None

        Include 'globals.inc'
        Include 'model.inc'

        Real*8 T,X(MaxNDE),XP(MaxNDE)
        Real*8 BWM, QCOM, V1M, V2M, Q1M, Q2M, CLtotM, VBM
        Real*8 BWR, QCOR, V1R, V2R, Q1R, Q2R, CLtotR, VBR
        Real*8 BWRB, QCORB, V1RB, V2RB, Q1RB, Q2RB, CLtotRB, VBRB
        Real*8 BWC, QCOC, V1C, V2C, Q1C, Q2C, CLtotC, VBC
        Real*8 BWD, QCOD, V1D, V2D, Q1D, Q2D, CLtotD, VBD
        Real*8 BWHO, QCOHO, V1HO, V2HO, Q1HO, Q2HO, CLtotHO, VBHO
        Real*8 BWH, QCOH, V1H, V2H, Q1H, Q2H, CLtotH, VBH
        Real*8 Kp1, Kp2, fd1, fd2, RB, FT, fdsum, Kpsum
        Real*8 fdsumtemp, fd1temp, FVTtemp, fd1_frac

CC
C-----------------------------------------------------------------------C
C   Enter Differential Equations Below  {e.g.  XP(1) = -P(1)*X(1) }    C
C----c------------------------------------------------------------------C

      fd1temp    = P(1)
      FVTtemp    = P(2)
      Kpsum      = P(3)

      fdsum = 1
      FT = FVTtemp/(1+FVTtemp)

      fd1 = fdsum * fd1temp/(1+fd1temp)
      fd2 = fdsum - fd1
      Kp1 = Kpsum
      Kp2 = Kpsum


      RB = 0.85

      BWM = 20
      QCOM = 8
      VBM = 1.7
      V2M = ( BWM-VBM )*(1-FT)!
      Q2M = QCOM * fd2
      V1M = ( BWM-VBM )*(FT)
      Q1M = QCOM * fd1
```

```
CLtotM =  P(4)!

BWR = 250
QCOR = 74
VBR = 13.5
V2R = ( BWR-VBR )*(1-FT)
Q2R = QCOR *fd2
V1R = ( BWR-VBR )*(FT)
Q1R = QCOR *fd1
CLtotR =  P(5)

BWRB = 2500
QCORB = 530
VBRB = 165
V2RB = ( BWRB-VBRB )*(1-FT)
Q2RB = QCORB * fd2
V1RB = ( BWRB-VBRB )*(FT)
Q1RB = QCORB * fd1
CLtotRB =  P(6)

BWC = 5000
QCOC = 445
VBC = 275
V2C = ( BWC-VBC )*(1-FT)
Q2C = QCOC * fd2
V1C = ( BWC-VBC )*(FT)
Q1C = QCOC * fd1
CLtotC =  P(7)

BWD = 10000
QCOD = 1200
VBD = 900
V2D = ( BWD-VBD )*(1-FT)
Q2D = QCOD * fd2
V1D = ( BWD-VBD )*(FT)
Q1D = QCOD * (fd1)
CLtotD = P(8)

BWHO = 530000
QCOHO = 39800
VBHO = 26000
V2HO = ( BWHO-VBHO )*(1-FT)
Q2HO = QCOHO * fd2
V1HO = ( BWHO-VBHO )*(FT)
Q1HO = QCOHO * (fd1)
```

```
        CLtotHO = P(9)

        BWH = 70000
        QCOH = 5600
        VBH = 5200
        V2H = (BWH - VBH) * (1-FT)
        Q2H = QCOH * fd2
        V1H = (BWH - VBH) * FT
        Q1H = QCOH * fd1
        CLtotH = P(10)

        XP(1) = ( - Q1HO*RB*(X(1)-X(2)/Kp1) - Q2HO*RB*(X(1)-X(3)/Kp2)
       & - CLtotHO*X(1) )/ VBHO / RB
        XP(2) = Q1HO*RB*(X(1)-X(2)/Kp1) / V1HO
        XP(3) = Q2HO*RB*(X(1)-X(3)/Kp2) / V2HO

        XP(4) = ( - Q1RB*RB*(X(4)-X(5)/Kp1) -Q2RB*RB*(X(4)-X(6)/Kp2)
       & - CLtotRB*X(4) ) / VBRB / RB
        XP(5) = Q1RB*RB*(X(4)-X(5)/Kp1) / V1RB
        XP(6) = Q2RB*RB*(X(4)-X(6)/Kp2) / V2RB

        XP(7) = ( - Q1R*RB*(X(7)-X(8)/Kp1) -Q2R*RB*(X(7)-X(9)/Kp2)
       & - CLtotR*X(7) ) / VBR / RB
        XP(8) = Q1R*RB*(X(7)-X(8)/Kp1) / V1R
        XP(9) = Q2R*RB*(X(7)-X(9)/Kp2) / V2R

        XP(10) = ( R(1) - Q1D*RB*(X(10)-X(11)/Kp1) - Q2D*RB*(X(10)
       & -X(12)/Kp2) - CLtotD*X(10) ) / VBD / RB
        XP(11) = Q1D*RB*(X(10)-X(11)/Kp1) / V1D
        XP(12) = Q2D*RB*(X(10)-X(12)/Kp2) / V2D

        XP(13) = ( - Q1C*RB*(X(13)-X(14)/Kp1) - Q2C*RB*(X(13)
       & -X(15)/Kp2) - CLtotC*X(13) ) / VBC / RB
        XP(14) = Q1C*RB*(X(13)-X(14)/Kp1) / V1C
        XP(15) = Q2C*RB*(X(13)-X(15)/Kp2) / V2C

        XP(16) = ( - Q1M*RB*(X(16)-X(17)/Kp1)
       & - Q2M*RB*(X(16)-X(18)/Kp2) - CLtotM*X(16)) / VBM / RB
        XP(17) = Q1M*RB*(X(16)-X(17)/Kp1) / V1M
        XP(18) = Q2M*RB*(X(16)-X(18)/Kp2) / V2M

        XP(19) = ( - Q1H*RB*(X(19)-X(20)/Kp1)
       & - Q2H*RB*(X(19)-X(21)/Kp2) - CLtotH*X(19)) / VBH / RB
        XP(20) = Q1H*RB*(X(19) - X(20)/Kp1) / V1H
        XP(21) = Q2H*RB*(X(19) - X(21)/Kp2) / V2H
```

```
C----------------------------------------------------------------------C
C----------------------------------------------------------------------C
C
      Return
      End

C######################################################################C

      Subroutine OUTPUT(Y,T,X)
      Implicit None

      Include 'globals.inc'
      Include 'model.inc'

      Real*8 Y(MaxNOE),T,X(MaxNDE)

CC
C----------------------------------------------------------------------C
C   Enter Output Equations Below   {e.g.  Y(1) = X(1)/P(2) }           C
C----c-----------------------------------------------------------------C

      Y(1) = X(1)
      Y(2) = X(4)
      Y(3) = X(7)
      Y(4) = X(10)
      Y(5) = X(13)
      Y(6) = X(16)
      Y(7) = X(19)

C----------------------------------------------------------------------C
C----------------------------------------------------------------------C
C
      Return
      End

C######################################################################C

      Subroutine VARMOD(V,T,X,Y)
      Implicit None

      Include 'globals.inc'
      Include 'model.inc'
```

```
        Real*8 V(MaxNOE),T,X(MaxNDE),Y(MaxNOE), Sigma, Intercept

CC
C----------------------------------------------------------------------C
C   Enter Variance Model Equations Below                               C
C         {e.g. V(1) = (PV(1) + PV(2)*Y(1))**2 }                       C
C----c-----------------------------------------------------------------C

      Sigma = PV(1)
      Intercept = PV(2)
      V(1) = ( Intercept + Sigma*Y(1) ) ** 2
      V(2) = ( Intercept + Sigma*Y(2) ) ** 2
      V(3) = ( Intercept + Sigma*Y(3) ) ** 2
      V(4) = ( Intercept + Sigma*Y(4) ) ** 2
      V(5) = ( Intercept + Sigma*Y(5) ) ** 2
      V(6) = ( Intercept + Sigma*Y(6) ) ** 2
      V(7) = ( Intercept + Sigma*Y(7) ) ** 2

C----------------------------------------------------------------------C
C----------------------------------------------------------------------C
C
        Return
        End

C######################################################################C

        Subroutine COVMOD(Pmean, ICmean, PC)
C  Defines any covariate model equations (MLEM, ITS)
        Implicit None

        Include 'globals.inc'
        Include 'model.inc'

        Real*8 PC(MaxNCP)
        Real*8 Pmean(MaxNSP+MaxNDE), ICmean(MaxNDE)

CC
C----------------------------------------------------------------------C
C     Enter # of Covariate Parameters                                  C
C----c-----------------------------------------------------------------C

        NCparam = 0    ! Enter # of Covariate Parameters.

CC
C----------------------------------------------------------------------C
C   Enter Symbol for Covariate Params {eg: PCsym(1)='CLRenal'}         C
```

```
C----c-----------------------------------------------------------------C


CC
C----------------------------------------------------------------------C
C   For the Model Params. that Depend on Covariates Enter the Equation C
C         {e.g. Pmean(1) =  PC(1)*R(2) }                               C
C----c-----------------------------------------------------------------C



C----------------------------------------------------------------------C
C----------------------------------------------------------------------C
C
        Return
        End

C######################################################################C

        Subroutine POPINIT(PmeanI,ICmeanI,PcovI,ICcovI, PCI)
C  Initial parameter values for population program parameters (ITS,
MLEM)

        Implicit None

        Include 'globals.inc'
        Include 'model.inc'

        Integer I,J
        Real*8 PmeanI(MaxNSP+MaxNDE), ICmeanI(MaxNDE)
        Real*8 PcovI(MaxNSP+MaxNDE,MaxNSP+MaxNDE), ICcovI(MaxNDE,MaxNDE)
        Real*8 PCI(MaxNCP)

CC
C----------------------------------------------------------------------C
C  Enter Initial Values for Population Means                           C
C          {  e.g. PmeanI(1) = 10.0    }                               C
C----c-----------------------------------------------------------------C


CC
C----------------------------------------------------------------------C
C   Enter Initial Values for Pop. Covariance Matrix (Lower Triang.)    C
C         {  e.g. PcovI(2,1) = 0.25    }                               C
C----c-----------------------------------------------------------------C
```

```
CC
C-----------------------------------------------------------------C
C   Enter Values for Covariate Model Parameters                   C
C         {  e.g. PCI(1) = 2.0    }                               C
C----c------------------------------------------------------------C


C-----------------------------------------------------------------C
C-----------------------------------------------------------------C
C
        Return
        End

C#################################################################C

        Subroutine PRIOR(Pmean,Pcov,ICmean,ICcov)
C  Parameter mean and covariance values for MAP estimation (ID,NPD,STS)
        Implicit None

        Include 'globals.inc'
        Include 'model.inc'

        Integer I,J
        Real*8 Pmean(MaxNSP+MaxNDE), ICmean(MaxNDE)
        Real*8 Pcov(MaxNSP+MaxNDE,MaxNSP+MaxNDE), ICcov(MaxNDE,MaxNDE)

CC
C-----------------------------------------------------------------C
C  Enter Nonzero Elements of Prior Mean Vector                    C
C          {  e.g. Pmean(1) = 10.0    }                           C
C----c------------------------------------------------------------C


CC
C-----------------------------------------------------------------C
C   Enter Nonzero Elements of Covariance Matrix (Lower Triang.)     C
C         {  e.g. Pcov(2,1) = 0.25    }                           C
C----c------------------------------------------------------------C


C-----------------------------------------------------------------C
C-----------------------------------------------------------------C
C
        Return
        End

C#################################################################C
```

```
        Subroutine SPARAM(PS,P,IC)
        Implicit None

        Include 'globals.inc'

        Real*8 PS(MaxNSECP), P(MaxNSP+MaxNDE), IC(MaxNDE)
        Real*8 fdsum,fd1,fd2,FT,Fdsumtemp,fd1temp,FVTtemp,fd1_frac

CC
C-----------------------------------------------------------------------C
C   Enter Equations Defining Secondary Paramters                        C
C           {  e.g.  PS(1) = P(1)*P(2)    }                             C
C----c------------------------------------------------------------------C
!      Fdsumtemp = P(10)
      fd1temp    = P(1)
      FVTtemp    = P(2)
      fdsum = 1


      FT = FVTtemp/(1+FVTtemp)
      fd1 = fdsum * P(1)/(1+P(1))
      fd2 = fdsum - fd1

      PS(1) = fd1
      PS(2) = FT
      PS(3) = fd2
      PS(4) = 1-FT

C-----------------------------------------------------------------------C
C-----------------------------------------------------------------------C
C
        Return
        End

C#######################################################################C

        Subroutine AMAT(A)
        Implicit None

        Include 'globals.inc'
        Include 'model.inc'

        Integer I,J
        Real*8 A(MaxNDE,MaxNDE)
```

```
      DO I=1,Ndeqs
         Do J=1,Ndeqs
            A(I,J)=0.0D0
         End Do
      End Do

CC
C-----------------------------------------------------------------------C
C   Enter non zero elements of state matrix  {e.g.  A(1,1) = -P(1) }   C
C----c------------------------------------------------------------------C


C-----------------------------------------------------------------------C
C-----------------------------------------------------------------------C
C
      Return
      End

C#######################################################################C
```

4 References

al-Meshal, M.A., el-Sayed, Y.M., al-Angary, A.A., and al-Dardiri, M.M. (1993). Effect of oral activated charcoal on propranolol pharmacokinetics following intravenous administration to rabbits. *J Clin Pharm Ther* 18(1)**,** 39–44. doi: 10.1111/j.1365-2710.1993.tb00564.x.

Al Shaker, H.A., Qinna, N.A., Badr, M., Al Omari, M.M.H., Idkaidek, N., Matalka, K.Z., et al. (2017). Glucosamine modulates propranolol pharmacokinetics via intestinal permeability in rats. *Eur J Pharm Sci* 105**,** 137–143. doi: 10.1016/j.ejps.2017.05.012.

Aramaki, S., Mori, M., Nakata, M., Shinohara, A., and Koizumi, T. (2000). Pharmacokinetics of propranolol and its metabolites in horses after intravenous or oral administration. *Biol Pharm Bull* 23(11)**,** 1333–1340. doi: 10.1248/bpb.23.1333.

Aravindh, S., and Jacob, N. (2021). Blood transfusion in animals: A review. *J Entomol Zool Stud* 9(5)**,** 357–361.

Arendt, R.M., Greenblatt, D.J., deJong, R.H., Bonin, J.D., and Abernethy, D.R. (1984). Pharmacokinetics, central nervous system uptake, and lipid solubility of propranolol, acebutolol, and sotalol. *Cardiology* 71(6)**,** 307–314. doi: 10.1159/000173684.

Aro, A., Anttila, M., Korhonen, T., and Sundquist, H. (1982). Pharmacokinetics of propranolol and sotalol in hyperthyroidism. *Eur J Clin Pharmacol* 21(5)**,** 373–377. doi: 10.1007/BF00542321.

Bano, G., Raina, R.K., Zutshi, U., Bedi, K.L., Johri, R.K., and Sharma, S.C. (1991). Effect of piperine on bioavailability and pharmacokinetics of propranolol and theophylline in healthy volunteers. *Eur J Clin Pharmacol* 41(6)**,** 615–617. doi: 10.1007/BF00314996.

Bargar, E.M., Walle, U.K., Bai, S.A., and Walle, T. (1983). Quantitative metabolic fate of propranolol in the dog, rat, and hamster using radiotracer, high performance liquid chromatography, and gas chromatography-mass spectrometry techniques. *Drug Metab Dispos* 11(3)**,** 266–272.

Bartorelli, C., and Gerola, A. (1963). Tidal Volume, Oxygen Uptake, Cardiac Output, and Body Surface in the Cat. *Am J Physiol* 205**,** 588–590. doi: 10.1152/ajplegacy.1963.205.3.588.

Bianchetti, G., Graziani, G., Brancaccio, D., Morganti, A., Leonetti, G., Manfrin, M., et al. (1976). Pharmacokinetics and effects of propranolol in terminal uraemic patients and in patients undergoing regular dialysis treatment. *Clin Pharmacokinet* 1(5)**,** 373–384. doi: 10.2165/00003088-197601050-00004.

Bleske, B.E., Welage, L.S., Rose, S., Amidon, G.L., and Shea, M.J. (1995). The effect of dosage release formulations on the pharmacokinetics of propranolol stereoisomers in humans. *J Clin Pharmacol* 35(4)**,** 374–378. doi: 10.1002/j.1552-4604.1995.tb04076.x.

Borgstrom, L., Johansson, C.G., Larsson, H., and Lenander, R. (1981). Pharmacokinetics of propranolol. *J Pharmacokinet Biopharm* 9(4)**,** 419–429. doi: 10.1007/BF01060886.

Bowman, S.L., Hudson, S.A., Simpson, G., Munro, J.F., and Clements, J.A. (1986). A comparison of the pharmacokinetics of propranolol in obese and normal volunteers. *Br J Clin Pharmacol* 21(5)**,** 529–532. doi: 10.1111/j.1365-2125.1986.tb02837.x.

Brown, R.P., Delp, M.D., Lindstedt, S.L., Rhomberg, L.R., and Beliles, R.P. (1997). Physiological parameter values for physiologically based pharmacokinetic models. *Toxicol Ind Health* 13(4)**,** 407–484. doi: 10.1177/074823379701300401.

Carmona, M.J., Malbouisson, L.M., Pereira, V.A., Bertoline, M.A., Omosako, C.E., Le Bihan, K.B., et al. (2005). Cardiopulmonary bypass alters the pharmacokinetics of propranolol in patients undergoing cardiac surgery. *Braz J Med Biol Res* 38(5)**,** 713–721. doi: 10.1590/S0100-879X2005000500008.

Charles, B.G., Ravenscroft, P.J., and Renshaw, P.J. (1982). Comparative pharmacokinetics of propranolol and 4-hydroxypropranolol using conventional and long-acting propranolol. *J*

Pharm Pharmacol 34(6), 403–404. doi: 10.1111/j.2042-7158.1982.tb04742.x.
Chen, J., Hsieh, Y., Cook, J., Morrison, R., and Korfmacher, W.A. (2006). Supercritical fluid chromatography-tandem mass spectrometry for the enantioselective determination of propranolol and pindolol in mouse blood by serial sampling. *Anal Chem* 78(4), 1212–1217. doi: 10.1021/ac0516178.
Cheung, S.Y.A., Rodgers, T., Aarons, L., Gueorguieva, I., Dickinson, G.L., Murby, S., et al. (2018). Whole body physiologically based modelling of beta-blockers in the rat: events in tissues and plasma following an i.v. bolus dose. *Br J Pharmacol* 175(1), 67–83. doi: 10.1111/bph.14071.
Chow, H.H., and Lalka, D. (1993). Pharmacokinetics of D-propranolol following oral, intra-arterial and intraportal administration: contrasting effects of oral glucose pretreatment. *Biopharm Drug Dispos* 14(3), 217–231. doi: 10.1002/bdd.2510140305.
Corbo, M., Wang, P.R., Li, J.K., and Chien, Y.W. (1989). Effect of propranolol on the myocardial contractility of normotensive and spontaneously hypertensive rabbits: relationship of pharmacokinetics and pharmacodynamics. *J Pharmacokinet Biopharm* 17(5), 551–570. doi: 10.1007/BF01071349.
Davies, B., and Morris, T. (1993). Physiological parameters in laboratory animals and humans. *Pharm Res* 10(7), 1093–1095. doi: 10.1023/a:1018943613122.
Dyke, T.M., Hubbell, J.A., Sams, R.A., and Hinchcliff, K.W. (1998). Hepatic blood flow in horses during the recuperative period from maximal exercise. *Am J Vet Res* 59(11), 1476–1480.
Fagerholm, U., Johansson, M., and Lennernas, H. (1996). Comparison between permeability coefficients in rat and human jejunum. *Pharm Res* 13(9), 1336–1342. doi: 10.1023/a:1016065715308.
Gangji, D., Juvent, M., Niset, G., Wathieu, M., Degreve, M., Bellens, R., et al. (1984). Study of the influence of nifedipine on the pharmacokinetics and pharmacodynamics of propranolol, metoprolol and atenolol. *Br J Clin Pharmacol* 17 Suppl 1(Suppl 1), 29S–35S. doi: 10.1111/j.1365-2125.1984.tb02425.x.
Gillespie, W.R., and Veng-Pedersen, P. (1985). Theorems and implications of a model-independent elimination/distribution function decomposition of linear and some nonlinear drug dispositions. II. Clearance concepts applied to the evaluation of distribution kinetics. *J Pharmacokinet Biopharm* 13(4), 441–451. doi: 10.1007/BF01061479.
Gomeni, R., Bianchetti, G., Sega, R., and Morselli, P.L. (1977). Pharmacokinetics of propranolol in normal healthy volunteers. *J Pharmacokinet Biopharm* 5(3), 183–192. doi: 10.1007/BF01065394.
Greenblatt, D.J., Scavone, J.M., Harmatz, J.S., Engelhardt, N., and Shader, R.I. (1993). Cognitive effects of beta-adrenergic antagonists after single doses: pharmacokinetics and pharmacodynamics of propranolol, atenolol, lorazepam, and placebo. *Clin Pharmacol Ther* 53(5), 577–584. doi: 10.1038/clpt.1993.73.
Henry, D., Brent, P., Whyte, I., Mihaly, G., and Devenish-Meares, S. (1987). Propranolol steady-state pharmacokinetics are unaltered by omeprazole. *Eur J Clin Pharmacol* 33(4), 369–373. doi: 10.1007/BF00637632.
Hitzenberger, G., Fitscha, P., Beveridge, T., Nuesch, E., and Pacha, W. (1982). Effects of age and smoking on the pharmacokinetics of pindolol and propranolol. *Br J Clin Pharmacol* 13(Suppl 2), 217S–222S. doi: 10.1111/j.1365-2125.1982.tb01914.x.
Hunt, B.A., Bottorff, M.B., Herring, V.L., Self, T.H., and Lalonde, R.L. (1990). Effects of calcium channel blockers on the pharmacokinetics of propranolol stereoisomers. *Clin Pharmacol Ther* 47(5), 584–591. doi: 10.1038/clpt.1990.79.
Jacobs, G., Whittem, T., Sams, R., Calvert, C., and Ferguson, D. (1997). Pharmacokinetics of propranolol in healthy cats during euthyroid and hyperthyroid states. *Am J Vet Res* 58(4),

398–403.
Jeong, Y.-S., and Jusko, W.J. (2021). Meta-Assessment of Metformin Absorption and Disposition Pharmacokinetics in Nine Species. *Pharmaceuticals* 14(6)**,** 545.
Jeong, Y.S., Kim, M.S., and Chung, S.J. (2022a). Determination of the Number of Tissue Groups of Kinetically Distinct Transit Time in Whole-Body Physiologically Based Pharmacokinetic (PBPK) Models I: Theoretical Consideration of Bottom-Up Approach of Lumping Tissues in Whole-Body PBPK. *AAPS J* 24(5)**,** 90. doi: 10.1208/s12248-022-00732-y.
Jeong, Y.S., Kim, M.S., and Chung, S.J. (2022b). Determination of the Number of Tissue Groups of Kinetically Distinct Transit Time in Whole-Body Physiologically Based Pharmacokinetic (PBPK) Models II: Practical Application of Tissue Lumping Theories for Pharmacokinetics of Various Compounds. *AAPS J* 24(5)**,** 91. doi: 10.1208/s12248-022-00733-x.
Jeong, Y.S., Yim, C.S., Ryu, H.M., Noh, C.K., Song, Y.K., and Chung, S.J. (2017). Estimation of the minimum permeability coefficient in rats for perfusion-limited tissue distribution in whole-body physiologically-based pharmacokinetics. *Eur J Pharm Biopharm* 115**,** 1–17. doi: 10.1016/j.ejpb.2017.01.026.
Johnson, J.A., and Burlew, B.S. (1992). Racial differences in propranolol pharmacokinetics. *Clin Pharmacol Ther* 51(5)**,** 495–500. doi: 10.1038/clpt.1992.53.
Komura, H., Kawase, A., and Iwaki, M. (2005). Application of substrate depletion assay for early prediction of nonlinear pharmacokinetics in drug discovery: assessment of nonlinearity of metoprolol, timolol, and propranolol. *J Pharm Sci* 94(12)**,** 2656–2666. doi: 10.1002/jps.20490.
Krediet, R.T., Dunning, A.J., and Offerhaus, L. (1980). Relationship of propranolol pharmacokinetics to antihypertensive effect and beta-adrenergic blockade in the treatment of hypertension. *Eur J Clin Pharmacol* 18(5)**,** 391–394. doi: 10.1007/BF00636790.
Laethem, M.E., Belpaire, F.M., Wijnant, P., and Bogaert, M.G. (1995). Stereoselective pharmacokinetics of oxprenolol, propranolol, and verapamil: species differences and influence of endotoxin. *Chirality* 7(8)**,** 616–622. doi: 10.1002/chir.530070811.
Laethem, M.E., Belpaire, F.M., Wijnant, P., Rosseel, M.T., and Bogaert, M.G. (1994). Influence of endotoxin on the stereoselective pharmacokinetics of oxprenolol, propranolol, and verapamil in the rat. *Chirality* 6(5)**,** 405–410. doi: 10.1002/chir.530060508.
Lautt, W.W. (1977). Effects of acute, passive hepatic congestion on blood flow and oxygen uptake in the intact liver of the cat. *Circ Res* 41(6)**,** 787–790. doi: 10.1161/01.res.41.6.787.
Leahey, W.J., Neill, J.D., Varma, M.P., and Shanks, R.G. (1980). Comparison of the efficacy and pharmacokinetics of conventional propranolol and a long acting preparation of propranolol. *Br J Clin Pharmacol* 9(1)**,** 33–40. doi: 10.1111/j.1365-2125.1980.tb04793.x.
Lee, Y.H., and Ku, Y.S. (1999). Effects of cyclosporin on the pharmacokinetics of propranolol after intravenous and oral administration to control rats and to rats with uranyl nitrate-induced acute renal failure. *J Pharm Pharmacol* 51(10)**,** 1149–1154. doi: 10.1211/0022357991776840.
Lennard, M.S., Jackson, P.R., Freestone, S., Tucker, G.T., Ramsay, L.E., and Woods, H.F. (1984). The relationship between debrisoquine oxidation phenotype and the pharmacokinetics and pharmacodynamics of propranolol. *Br J Clin Pharmacol* 17(6)**,** 679–685. doi: 10.1111/j.1365-2125.1984.tb02403.x.
Lennernas, H. (1997). Human jejunal effective permeability and its correlation with preclinical drug absorption models. *J Pharm Pharmacol* 49(7)**,** 627–638. doi: 10.1111/j.2042-7158.1997.tb06084.x.
Levy, A., Ngai, S.H., Finck, A.D., Kawashima, K., and Spector, S. (1976). Disposition of propranolol isomers in mice. *Eur J Pharmacol* 40(1)**,** 93–100. doi: 10.1016/0014-2999(76)90358-7.

Lowenthal, D.T., Briggs, W.A., Gibson, T.P., Nelson, H., and Cirksena, W.J. (1974). Pharmacokinetics of oral propranolol in chronic renal disease. *Clin Pharmacol Ther* 16(5 Part 1)**,** 761–769. doi: 10.1002/cpt1974165part1761.
McCourty, J.C., Silas, J.H., Tucker, G.T., and Lennard, M.S. (1988). The effect of combined therapy on the pharmacokinetics and pharmacodynamics of verapamil and propranolol in patients with angina pectoris. *Br J Clin Pharmacol* 25(3)**,** 349–357. doi: 10.1111/j.1365-2125.1988.tb03313.x.
O'Hare, M.F., Kinney, C.D., Murnaghan, G.A., and McDevitt, D.G. (1984). Pharmacokinetics of propranolol during pregnancy. *Eur J Clin Pharmacol* 27(5)**,** 583–587. doi: 10.1007/BF00556896.
Olanoff, L.S., Walle, T., Walle, U.K., Cowart, T.D., and Gaffney, T.E. (1984). Stereoselective clearance and distribution of intravenous propranolol. *Clin Pharmacol Ther* 35(6)**,** 755–761. doi: 10.1038/clpt.1984.107.
Poulin, P., and Theil, F.P. (2002). Prediction of pharmacokinetics prior to in vivo studies. II. Generic physiologically based pharmacokinetic models of drug disposition. *J Pharm Sci* 91(5)**,** 1358–1370. doi: 10.1002/jps.10128.
Rodgers, T., Leahy, D., and Rowland, M. (2005). Tissue distribution of basic drugs: accounting for enantiomeric, compound and regional differences amongst beta-blocking drugs in rat. *J Pharm Sci* 94(6)**,** 1237–1248. doi: 10.1002/jps.20323.
Routledge, P.A., and Shand, D.G. (1979). Clinical pharmacokinetics of propranolol. *Clin Pharmacokinet* 4(2)**,** 73–90. doi: 10.2165/00003088-197904020-00001.
Schaefer, H.G., Harrison, D., Hocking, M.P., Limberg, J., and Derendorf, H. (1990). Effects of truncal vagotomy and partial gastrectomy on the pharmacokinetics of propranolol enantiomers in dogs. *J Pharm Sci* 79(4)**,** 292–294. doi: 10.1002/jps.2600790404.
Schoors, D.F., Vercruysse, I., Musch, G., Massart, D.L., and Dupont, A.G. (1990). Influence of nicardipine on the pharmacokinetics and pharmacodynamics of propranolol in healthy volunteers. *Br J Clin Pharmacol* 29(5)**,** 497–501. doi: 10.1111/j.1365-2125.1990.tb03671.x.
Serlin, M.J., Orme, M.L., MacIver, M., Green, G.J., Sibeon, R.G., and Breckenridge, A.M. (1983). The pharmacodynamics and pharmacokinetics of conventional and long-acting propranolol in patients with moderate hypertension. *Br J Clin Pharmacol* 15(5)**,** 519–527. doi: 10.1111/j.1365-2125.1983.tb02085.x.
Shibasaki, S., Asahina, M., Kawamata, Y., Kojo, M., Nishigaki, R., and Umemura, K. (1989). The inhibitory effects of cimetidine on elimination and distribution of propranolol in rats. *J Pharmacobiodyn* 12(9)**,** 549–557. doi: 10.1248/bpb1978.12.549.
Shiga, T., Fujimura, A., Tateishi, T., Ohashi, K., and Ebihara, A. (1993). The effect of age on diurnal variation in the pharmacokinetics of propranolol in hypertensive subjects. *Eur J Clin Pharmacol* 44(5)**,** 489–492. doi: 10.1007/BF00315550.
Singh, K., Tripp, S.L., Dunton, A.W., Douglas, F.L., and Rakhit, A. (1991). Determination of in vivo hepatic extraction ratio from in vitro metabolism by rat hepatocytes. *Drug Metab Dispos* 19(5)**,** 990–996.
Smith, M.T., Livingstone, I., Eadie, M.J., Hooper, W.D., and Triggs, E.J. (1983). Chronic propranolol administration during pregnancy. Maternal pharmacokinetics. *Eur J Clin Pharmacol* 25(4)**,** 481–490. doi: 10.1007/BF00542115.
Straka, R.J., Lalonde, R.L., Pieper, J.A., Bottorff, M.B., and Mirvis, D.M. (1987). Nonlinear pharmacokinetics of unbound propranolol after oral administration. *J Pharm Sci* 76(7)**,** 521–524. doi: 10.1002/jps.2600760706.
Taegtmeyer, A.B., Haschke, M., Tchambaz, L., Buylaert, M., Tschopl, M., Beuers, U., et al. (2014). A study of the relationship between serum bile acids and propranolol pharmacokinetics and

pharmacodynamics in patients with liver cirrhosis and in healthy controls. *PLoS One* 9(6)**,** e97885. doi: 10.1371/journal.pone.0097885.

Tateishi, T., Nakashima, H., Shitou, T., Kumagai, Y., Ohashi, K., Hosoda, S., et al. (1989). Effect of diltiazem on the pharmacokinetics of propranolol, metoprolol and atenolol. *Eur J Clin Pharmacol* 36(1)**,** 67–70. doi: 10.1007/BF00561026.

Tawara, K., Kawashima, K., Ishikawa, H., Yamamoto, K., Saito, K., Ebihara, A., et al. (1981). Pharmacokinetics and pharmacodynamics of propranolol stereoisomers in hyperthyroid patients. *Eur J Clin Pharmacol* 19(3)**,** 197–203. doi: 10.1007/BF00561949.

Taylor, E.A., and Turner, P. (1981). The distribution of propranolol, pindolol and atenolol between human erythrocytes and plasma. *Br J Clin Pharmacol* 12(4)**,** 543–548. doi: 10.1111/j.1365-2125.1981.tb01263.x.

Terao, N., and Shen, D.D. (1984). Pharmacokinetics of l-propranolol during repetitive dosing in normal and uranyl nitrate-induced renal failure rats. *J Pharmacokinet Biopharm* 12(5)**,** 479–493. doi: 10.1007/BF01060127.

Vanakoski, J., and Seppala, T. (1995). Effects of a Finnish sauna on the pharmacokinetics and haemodynamic actions of propranolol and captopril in healthy volunteers. *Eur J Clin Pharmacol* 48(2)**,** 133–137. doi: 10.1007/BF00192738.

Veng-Pedersen, P. (1984). Theorems and implications of a model independent elimination/distribution function decomposition of linear and some nonlinear drug dispositions. I. Derivations and theoretical analysis. *J Pharmacokinet Biopharm* 12(6)**,** 627–648. doi: 10.1007/BF01059557.

Vercruysse, I., Schoors, D.F., Massart, D.L., and Dupont, A.G. (1993). Influences of the calcium antagonists nicardipine and nifedipine, and the calcium agonist BAY-K-8644, on the pharmacokinetics of propranolol in rats. *Cardiovasc Drugs Ther* 7(4)**,** 721–726. doi: 10.1007/BF00877826.

Vermeulen, A.M., Belpaire, F.M., Moerman, E., de Smet, F., and Bogaert, M.G. (1992). The influence of aging on the stereoselective pharmacokinetics of propranolol in the rat. *Chirality* 4(2)**,** 73–79. doi: 10.1002/chir.530040202.

Walle, U.K., Thibodeaux, H., Privitera, P.J., and Walle, T. (1989). Stereochemistry of tissue distribution of racemic propranolol in the dog. *Chirality* 1(3)**,** 192–196. doi: 10.1002/chir.530010303.

Wang, Y., Wang, Z., Zuo, Z., Tomlinson, B., Lee, B.T., Bolger, M.B., et al. (2013). Clinical pharmacokinetics of buffered propranolol sublingual tablet (Promptol)-application of a new "physiologically based" model to assess absorption and disposition. *AAPS J* 15(3)**,** 787–796. doi: 10.1208/s12248-013-9479-1.

Weiss, Y.A., Safar, M.E., Chevillard, C., Frydman, A., Simon, A., Lemaire, P., et al. (1976). Comparison of the pharmacokinetics of intravenous dl-propranolol in borderline and permanent hypertension. *Eur J Clin Pharmacol* 10(6)**,** 387–393. doi: 10.1007/BF00563074.

Wenbin, L., Rong, W., Hua, X., Juanhong, Z., Xiaoyu, W., and Zhengping, J. (2015). Effects on Pharmacokinetics of Propranolol and Other Factors in Rats After Acute Exposure to High Altitude at 4,010 m. *Cell Biochem Biophys* 72(1)**,** 27–36. doi: 10.1007/s12013-014-0397-3.

Williams, F.M., Leeser, J.E., and Rawlins, M.D. (1986). Pharmacodynamics and pharmacokinetics of single doses of ketanserin and propranolol alone and in combination in healthy volunteers. *Br J Clin Pharmacol* 22(3)**,** 301–308. doi: 10.1111/j.1365-2125.1986.tb02891.x.

Wojcicki, J., Jaroszynska, M., Drozdzik, M., Pawlik, A., Gawronska-Szklarz, B., and Sterna, R. (2003). Comparative pharmacokinetics and pharmacodynamics of propranolol and atenolol in normolipaemic and hyperlipidaemic obese subjects. *Biopharm Drug Dispos* 24(5)**,** 211–218.

doi: 10.1002/bdd.357.
Wojcicki, J., Sulzyc-Bielicka, V., Kutrzeba, J., Gawronska-Szklarz, B., Drozdzik, M., and Sterna, Z. (1999). Studies on the pharmacokinetics and pharmacodynamics of propranolol in hyperlipidemia. *J Clin Pharmacol* 39(8)**,** 826–833. doi: 10.1177/00912709922008498.
Xie, H.G., and Chen, X. (1995). Sex differences in pharmacokinetics of oral propranolol in healthy Chinese volunteers. *Zhongguo Yao Li Xue Bao* 16(5)**,** 468–470.
Yasuhara, M., Yatsuzuka, A., Yamada, K., Okumura, K., Hori, R., Sakurai, T., et al. (1990). Alteration of propranolol pharmacokinetics and pharmacodynamics by quinidine in man. *J Pharmacobiodyn* 13(11)**,** 681–687. doi: 10.1248/bpb1978.13.681.
Yoshigae, Y., Imai, T., Aso, T., and Otagiri, M. (1998). Species differences in the disposition of propranolol prodrugs derived from hydrolase activity in intestinal mucosa. *Life Sci* 62(14)**,** 1231–1241. doi: 10.1016/s0024-3205(98)00053-8.
